# Role of support bio-templating in Ni/Al_2_O_3_ catalysts for hydrogen production via dry reforming of methane

**DOI:** 10.1038/s41598-023-43782-8

**Published:** 2023-10-09

**Authors:** Tayebeh Roostaei, Mohammad Reza Rahimpour

**Affiliations:** Department of Chemical Engineering, Shiraz University, Shiraz, Iran

**Keywords:** Chemical engineering, Catalyst synthesis

## Abstract

Bio-templating, a synthetic approach inspired by nature, is an emerging area in material engineering. In this study, waste leaves of Sycamore were utilized as a bio-template for producing alumina support to prepare catalyst. The performance of Ni and Ce impregnated on bio-templated alumina support was investigated in dry reforming of methane for the first time. The effect of process and catalytic variables were examined in detail. The results showed that impregnation of 20% Ni and 3% Ce on the bio-templated alumina led to improved Ni dispersion and achieving the maximum CH_4_ conversion of 88.7%, CO_2_ conversion of 78.5%, and H_2_ yield of 85.3%, compared to 84.4%, 75.6% and 83.4% for the non-templated catalyst at 700 °C, respectively. Detailed characterization of the catalysts revealed that the enhanced performance in the bio-templated catalyst could be attributed to smaller Ni particles, superior dispersion of Ni on the support, the mesoporous structure of alumina, and the larger surface area of support. Furthermore, analysis of the used catalyst showed reduced coke formation on the catalyst surface and high stability of bio-templated catalysts, highlighting the main advantage of bio-templated catalysts over non-templated ones. The findings presented in this study contribute to the potential future applications of bio-templating materials and shed light on the rational design of bio-templating materials.

## Introduction

Methane (CH_4_) and carbon dioxide (CO_2_) are two potent greenhouse gases (GHGs) with detrimental impact on the environment, which directly contributes to climate change^1^. Increasing the GHGs emission has prompted the search for solutions to address this environmental crisis^2^. From a carbon economy perspective, transforming such unfavorable gases into value-added products has gained much attention in environmental research^3–6^. Accordingly, converting CH_4_ and CO_2_ into syngas (H_2_ and CO), known as dry reforming of methane (DRM), has proven to be multi-dimensional beneficial^7^. The main reason is the environmentally friendly nature of hydrogen as an efficient energy carrier. In addition, hydrogen is a critical intermediate in producing several strategic products, such as methanol, acetic acid, and ammonia^4,8^. DRM, as presented in Eq. (1), emerges as an economical alternative compared to other reforming methods, mostly due to the elimination of the separation step at the end of the process^9,10^. Aside from the mentioned benefits of DRM, it is a promising pathway for carbon capture and utilization (CCU)^11–13^. If all hydrogen produced annually (currently 60Mt year^–1^) were supplied through DRM instead of steam reforming, approximately 0.5Gt year^–1^ of CO_2_ would be cut off immediately, approaching the 2030 target in a decarbonization roadmap^14,15^. The DRM produces syngas with an H_2_/CO molar ratio close to unity, which is favourable to generate valuable chemicals, such as Fischer–Tropsch process and ammonia production^16–18^.

1 $${\text{DRM}}: {\text{CH}}_{{4}} + {\text{CO}}_{{2}} \leftrightarrow {\text{2CO}} + {\text{2H}}_{{2}} \quad \quad {\Delta H}_{{298{\text{ K}}}}^{ \circ } = 247{\text{ kJmol}}^{ - 1}$$

Nevertheless, the main challenge of DRM is carbon accumulation on catalyst’s surface, which occurs at high reaction temperatures of an endothermic DRM process^19^. Catalyst deactivation arises from undesired side reactions of CH_4_ decomposition, Boudouard reaction, and CO reduction^20,21^. Hence, several studies have been done on developing a catalyst to prevent or minimize carbon formation and catalyst deactivation.

Among various examined catalysts for the DRM process, noble metals like Ru^22^, Pt^23^, Pd^24^, and Rh^25^ showed reduced coke formation and excellent performance in the DRM. Though, the problems associated with these metals are their high cost, unavailability, and low stability in high reaction temperatures of DRM^26,27^. In contrast, the supported transition metals, especially Ni, have been extensively used as a potential alternative because of their high initial activity and relatively low cost^27–31^. However, the catalyst deactivation hindered the Ni-based DRM commercialization.

To improve the stability of DRM catalysts, many approaches have been focused on evaluating different parameters, including reducing Ni particle size, dispersing Ni particles on support, employing highly porous support, and adding a metal oxide as a promoter, such as Ce^32–34^, Y^35^, La^36^, Mg^27,37,38^, and B^19^ to oxidize the formed coke on the catalysts’ surface.

High Ni dispersion could be achieved by trapping the metal nanoparticles inside the mesoporous structure of support. Consequently, the performance of the DRM catalyst strongly depends on the supporting material. Appropriate support must also resist high temperatures and maintain metal dispersion during the reaction. Despite numerous efforts by researchers, developing a catalyst with excellent resistance against coke formation for the DRM process remains a significant challenge.

One approach to enhance the supporting material surface area and increase the structure porosity is using a templating material in the catalyst support preparation. In this line, the choice of template plays a crucial role in obtaining efficient material with the desired properties. This template could be synthetic materials, such as nanoparticles, porous materials, and surface-active materials, or natural materials, like DNA, proteins, butterfly wings, and leaves. The later green templates derived from biological species, called bio-templates, are more desired in preparing metal oxide catalysts since they are inexpensive, non-polluting, sustainable, mechanically and chemically adaptable, and naturally abundant^39,40^. Using green templates in synthesizing catalysts is a sustainable route toward clean materials production by reducing the use of harmful materials and meanwhile having a porous structure with a high surface area. Moreover, several articles have been published on synthesizing bio-template materials^26–28,41–43^, but there are fewer reports on the application of these catalysts. Herein, we used Sycamore waste leaves, with a highly porous structure, as the bio-template to prepare alumina support. Sycamore is a local abundant tree in Iran and its leaves fall easily during autumn. The management of these bio-wastes is a major challenge which was tried to address in the present study.

This study investigates the potential of a novel green alumina, prepared by the bio-template method using waste leaves, as suitable supporting material for Ni active sites in the DRM process for the first time. The efficiencies of bio-templated and non-templated catalysts were compared, supported by appropriate characterization techniques. Various catalytic and processing parameters, including the amount of active phase, promoter loading, and reaction temperature, were examined. The results demonstrated the sintering and coke formation of the bio-templated catalysts were slightly reduced compared to the non-templated catalysts, leading to improved performance, and higher stability in long-term DRM reaction. To the authors' best knowledge, there is no report on the application of leaf-based catalysts in the DRM reaction. As a result, the findings of this study exemplify the successful application of bio-template materials through the rational design of an effective catalyst.

## Experimental

### Materials

Aluminum triisopropylate (Al[OCH(CH_3_)_2_]_3_), nickel (II)-nitrate hexahydrate (Ni(NO_3_)_2_)0.6H_2_O), cerium (III)-nitrate hexahydrate (Ce(NO_3_)_3_·6H_2_O), hydrochloric acid (37 wt%), and ethanol (> 98) were provided by Merck and used without further purification. Waste leaves of the Sycamore were collected from the local campus in Iran and used as a bio-template (Fig. S1).

### Pre-treatment of bio-template

**Figure 1:**
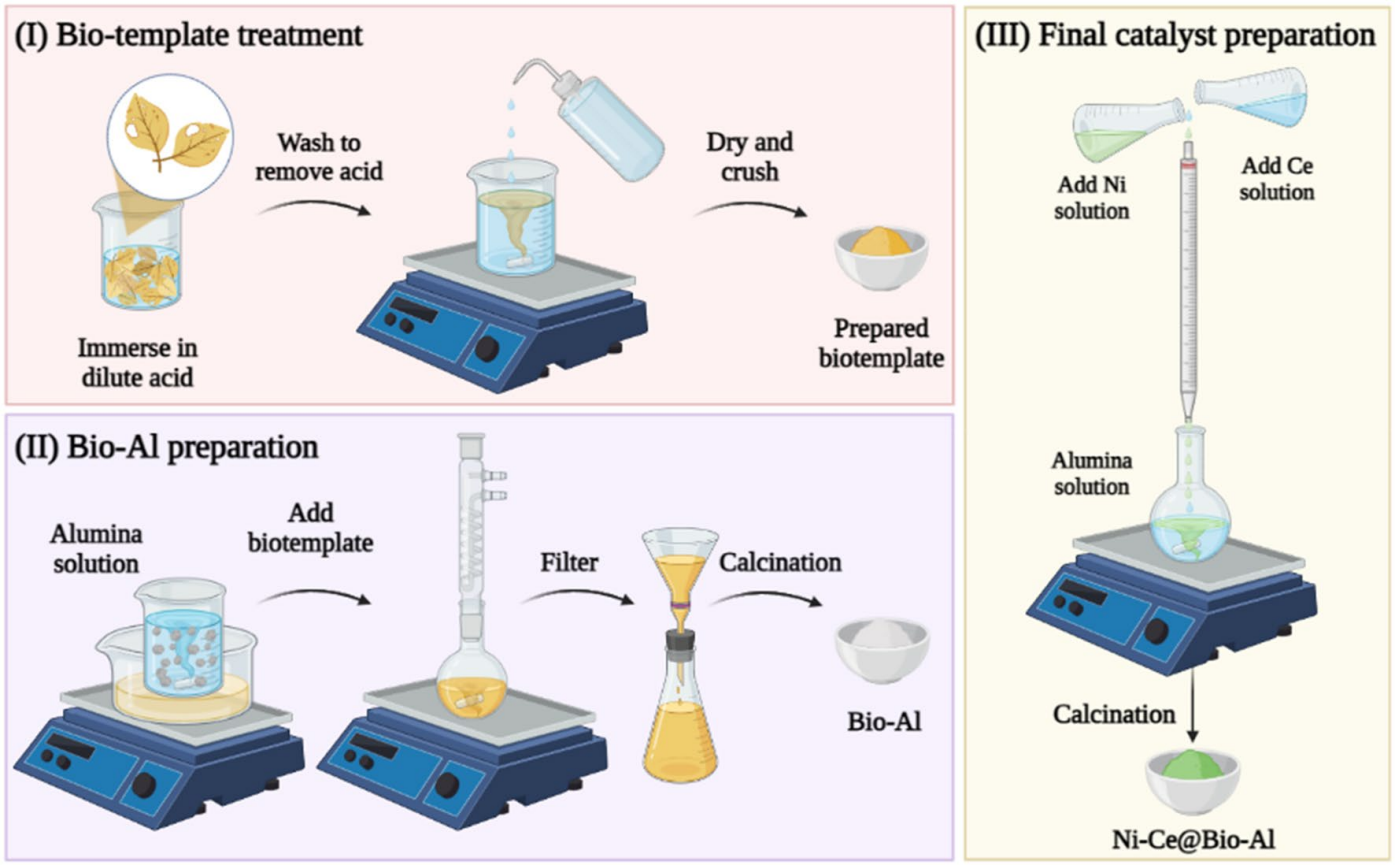
A schematic of Ni–Ce@Bio-Al preparation process: (I) preparation of bio-template, (II) preparation of Bio-Al support, (III) co-impregnating of Ni and Ce on Bio-Al to prepare the final catalyst.

The waste leaves were initially pre-treated to remove contaminants and get rid of inorganic ions. To this end, the leaves were immersed in dilute acid (5% v/v HCl) overnight. Next, they were washed with deionized water several times to remove acid, and then dried in an oven for 24 h. Consequently, the treated leaves were crushed using a mini-laboratory electric grinder to form a fine yellow powder (Step I in Fig. 1).

### Synthesis of final catalyst

The alumina support was prepared by the sol–gel method. For this purpose, 4 g Al[OCH(CH_3_)_2_]_3_ precursor salt was initially solved in 150 ml acidic water (pH = 4) and stirred for 3 h at 80 °C, followed by standing at the same temperature for 12 h. Subsequently, 2 g of the treated waste leaves were soaked in alumina precursor solution and stayed at 80 °C overnight. Next, the solution was filtered and dried in an oven at 100 °C for 30 min and calcined in a furnace at 800 °C for 3 h under air. The obtained white powder is designated as “Bio-Al” in the text (Step II in Fig. 1). To investigate the effect of bio-templating, a non-templated (bulk) alumina sample was prepared using the same procedure, except adding the bio-template. The non-templated Al_2_O_3_ was named Bulk-Al in the manuscript.

The wet impregnation method was employed to introduce the active phase (Ni) and promoter (Ce) to the prepared alumina. For this purpose, separated aqueous solutions of Ce(NO_3_)_2_.6H_2_O and Ni(NO_3_)_2_.6H_2_O with 0.05 M concentration were added dropwise to the suspension of prepared Bio-Al in the water simultaneously while stirring at 40 °C for 4 h. The prepared solid pastes were then kept in an oven at 100 °C for 15 h. Finally, the obtained samples were calcined at 650 °C for 3 h at a rate of 4 °C/min (Step III in Fig. 1). The calcined catalysts were designated as (z)Ni-(x)Ce@Bio-Al for bio-templated catalysts and (z)Ni-(x)Ce@Bulk-Al and in the text, in which z is Ni weight percentage with 10, 15, 20 and 25 wt% amounts, and x is Ce weight percentage with 0, 1.5, 3, and 4.5 wt% loadings. The amount of Ni and Ce loading ranges were chosen based on literature review^37,44,45^. Figure 1 presents a schematic of catalyst preparation steps.

### Catalyst characterization

Power X-ray diffraction (XRD, PHILIPS X’Pert PW1730) with a Cu-Kα monochromatizer (40 kV, 30 mA) and a Cu anode X-ray tube was used to analyze the crystalline phases of the samples. The catalyst morphology and structure were characterized by a field-emission scanning electron microscope (FESEM, TESCAN MI RA III) and a transmission electron microscope (TEM, Philips CM120). Energy-dispersive X-ray spectroscopy (EDX) analysis was used with a SAMX detector coupled with the FESEM instrument to measure the elemental composition of samples and their dispersions. Nitrogen adsorption–desorption isotherms were obtained using a surface area and porosity analyzer BELSORP MINI II at 200 °C. The Brunauer–Emmett–Teller (BET) methods and Barrett-Joyner-Halenda (BJH) models were used to calculate the specific surface areas of the mesopores and pore-size distributions, respectively. Temperature-programmed hydrogen reduction (H_2_-TPR, NanoSORD NS9) was used to study the reducibility of catalysts. The thermogravimetric analysis (TGA) technique was used to determine the thermal stability and amount of deposited coke on the synthesized catalysts with the aim of an SDT Q 600 instrument. Raman analysis was carried out to get information about the type of deposited coke using Raman spectrometer (Technooran company- model Ram-532-004). The inductively coupled plasma optical emission spectrometry (ICP-OES) technique was used to analyze the chemical compositions of final catalysts (VISTA-PRO spectrophotometer instrument).

### Catalyst activity in dry reforming of methane

**Figure 2:**
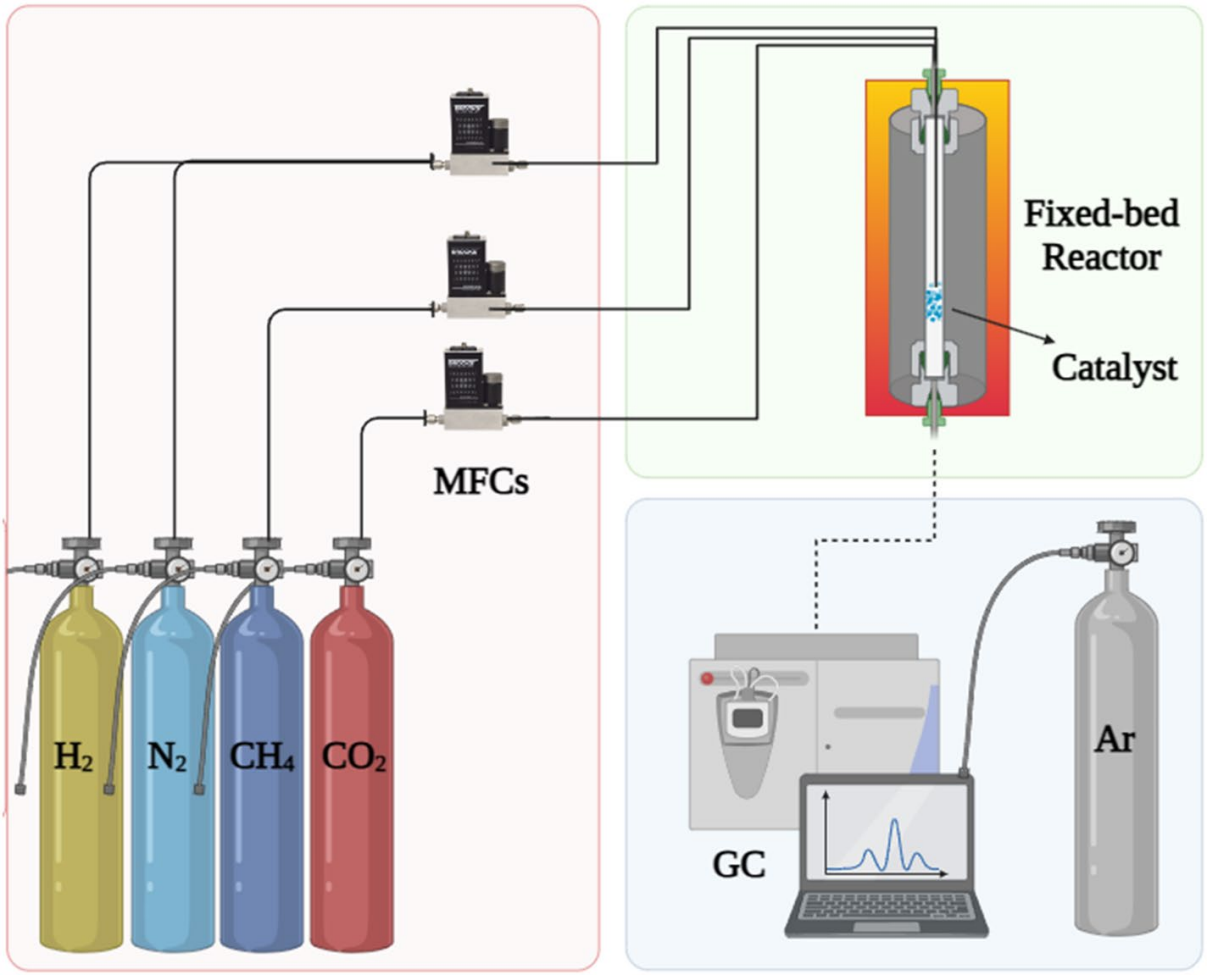
Schematic of experimental DRM set-up.

To determine the effectiveness of the prepared catalysts in the DRM process, practical tests were carried out at atmospheric pressure using an isothermal stainless steel fixed-bed reactor with a diameter of 1.6 cm and length of 1 m (Fig. S2). An electric furnace was used to control the reaction temperature. In a typical experiment, 1 g of the synthesized catalyst (catalyst size: 150–200 µm) was loaded into the reactor. Subsequently, the catalyst was reduced by elevating the reactor temperature to 750 °C for a duration of 1 h, using pure hydrogen with a flow rate of 150 ml/min. In the next step, the reactor temperature was fixed to the reaction temperature (600, 650, and 700 °C) and pure nitrogen was passed till reaching this temperature. CH_4_ and CO_2_ were then introduced into the reactor by the ratio of unity following the reaction’s stoichiometry, and a flow rate of 100 ml.min^-1^ with the constant gas hourly space velocity (GHSV) of 12,000 ml g_cat_^−1^ h^−1^ for 1 h. Six injections of an online Bruker 450 gas chromatograph (GC) instrument with a TCD were taken to analyze the composition of the exited dry gas stream at each reaction temperature. The TDX-01 column was used for gas separation and argon was applied as a carrier gas. A schematic presentation of the set-up is shown in Fig. 2.

To evaluate the catalysts’ performance, CH_4_ conversion (${\mathrm{X}}_{{\mathrm{CH}}_{4}}$), CO_2_ conversion (${\mathrm{X}}_{{\mathrm{CO}}_{2}}$), H_2_ yield (${\mathrm{Y}}_{{\mathrm{H}}_{2}}$), and H_2_/CO ratio were calculated using Eqs. (2)–(5), respectively ^46,47^.

2 $${\mathrm{X}}_{{\mathrm{CH}}_{4}}=\frac{ {\mathrm{F}}_{{\mathrm{CH}}_{4,\mathrm{in}}}-{\mathrm{F}}_{{\mathrm{CH}}_{4,\mathrm{out}}}}{{\mathrm{F}}_{{\mathrm{CH}}_{4,\mathrm{in}}}}\times 100$$

3 $${\mathrm{X}}_{{\mathrm{CO}}_{2}}=\frac{ {\mathrm{F}}_{{\mathrm{CO}}_{2,\mathrm{in}}}-{\mathrm{F}}_{{\mathrm{CO}}_{2,\mathrm{out}}}}{{\mathrm{F}}_{{\mathrm{CO}}_{2,\mathrm{in}}}}\times 100$$

4 $${\mathrm{Y}}_{{\mathrm{H}}_{2}}=\frac{{\mathrm{F}}_{{\mathrm{H}}_{2},\mathrm{out}}}{{2{\mathrm{F}}_{{\mathrm{CH}}_{4}},}_{\mathrm{in}}}\times 100$$

5 $$\frac{{\mathrm{H}}_{2}}{\mathrm{CO}}=\frac{{\mathrm{F}}_{{\mathrm{H}}_{2},\mathrm{out}}}{{\mathrm{F}}_{\mathrm{CO},\mathrm{out}}}$$

where ${\mathrm{F}}_{{\mathrm{CH}}_{4}}$, ${\mathrm{F}}_{{\mathrm{CO}}_{2}}$,${\mathrm{F}}_{{\mathrm{H}}_{2}}$, and ${\mathrm{F}}_{\mathrm{CO}}$ refer to CH_4,_ CO_2_, H_2_, and CO molar flow rates, and in and out subscripts denote input and output streams, respectively.

### Thermodynamic study

The thermodynamic analysis was conducted to understand the operating conditions in DRM and compare the theoretical values with the experimental ones. Predicting the equilibrium conversions is helpful to determine which reaction is complete and the extremum amount of each product^48^. The DRM reaction (Eq. 1) was considered as the main reaction and three other reactions, including reverse water–gas shift reaction (Eq. 6), methane cracking (Eq. 7), and CO disproportion (Eq. 8), were considered as the side reactions^49^.

6 $${\text{Reverse water}}-{\text{gas shift}}:{\text{CO}}_{2} + {\text{H}}_{2} \leftrightarrow {\text{CO}} + {\text{H}}_{2} {\text{O }}\quad\quad\Delta {\text{H}}_{{298{\text{K}}}}^{^\circ } = 41{\text{ kJ mol}}^{ - 1}$$

7 $${\text{Methane cracking }}:{\text{CH}}_{4} \leftrightarrow {\text{C}} + 2{\text{H}}_{2} { }\quad\quad\Delta {\text{H}}_{{298{\text{K}}}}^{^\circ } = 75{\text{ kJ mol}}^{ - 1}$$

8 $${\text{CO disproportion}}:2{\text{CO}} \leftrightarrow {\text{C}} + {\text{CO}}_{2} { }\quad\quad\Delta {\text{H}}_{{298{\text{K}}}}^{^\circ } = - 175{\text{ kJ mol}}^{ - 1}$$

The equilibrium thermodynamic calculations were carried out using Aspen Plus software Version 12.1. The thermodynamic model of Peng-Robinson thermodynamic was chosen for our system. Reactions 1, 6–8 with feed ratio (CO_2_/CH_4_) of unity and pressure of 1 bar were considered in this simulation. Temperature was set from 600 to 700 °C using sensitive analysis.

## Results and discussion

### Activity tests in dry reforming of methane

**Figure 3:**
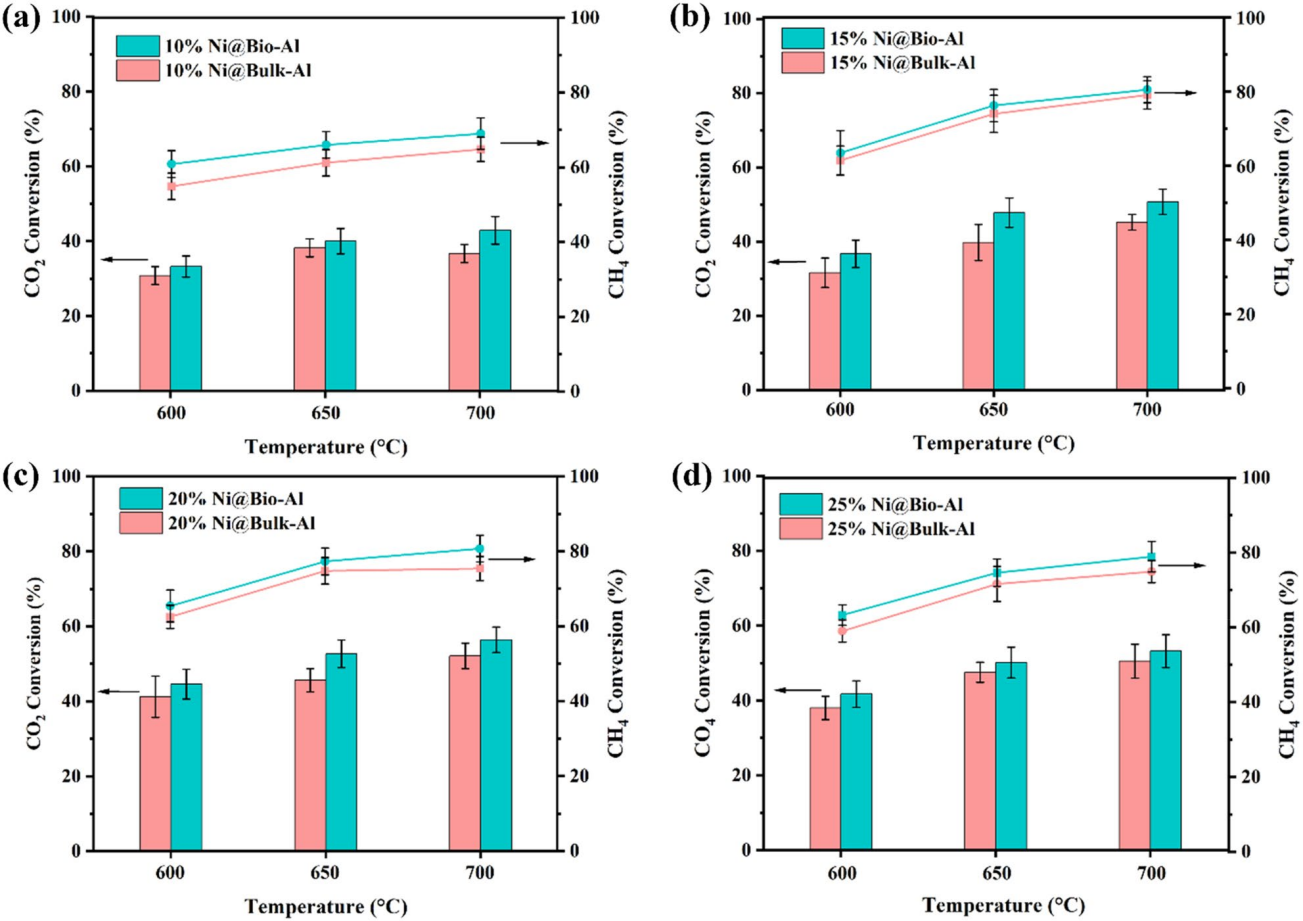
CO_2_ and CH_4_ conversion for (**a**) 10% Ni, (**b**) 15% Ni, (**c**) 20% Ni, and (**d**) 25% Ni loading on bio-templated and non-templated catalysts (without Ce).

To investigate the effect of support bio-templating on DRM performance and optimize the Ni and Ce percentages, the prepared catalysts with and without bio-template were tested at three temperatures (600, 650, and 700 °C). CH_4_ and CO_2_ conversion, H_2_ yield, and H_2_/CO ratio were calculated according to Eqs. (2)–(5), respectively. The results of CH_4_ and CO_2_ conversion for 10, 15, 20, and 25% Ni loading are shown in Fig. 3a to d. It is noteworthy that the promoter had not added at this stage. The results indicate a clear increasing trend of CH_4_ and CO_2_ conversion by enhancing the reaction temperature for all samples, regardless of the support structure and Ni loading amount. This can be attributed to the endothermic nature of DRM (Eq. 1), meaning that raising the temperature enhances the equilibrium conversion and leads to higher CH_4_ and CO_2_ conversion^30,50^. Furthermore, at higher temperatures, molecular collisions are more effective, further facilitating the reaction. For instance, by boosting the temperature from 600 to 700 °C for 25% Ni loading, the CH_4_ and CO_2_ conversion were increased by 25.2% and 27.5% for the bio-templated and 26.9% and 32.9% for the non-templated catalysts. Higher CH_4_ conversion than CO_2_ conversion is due to reverse water gas reaction (Eq. 6).

**Figure 4:**
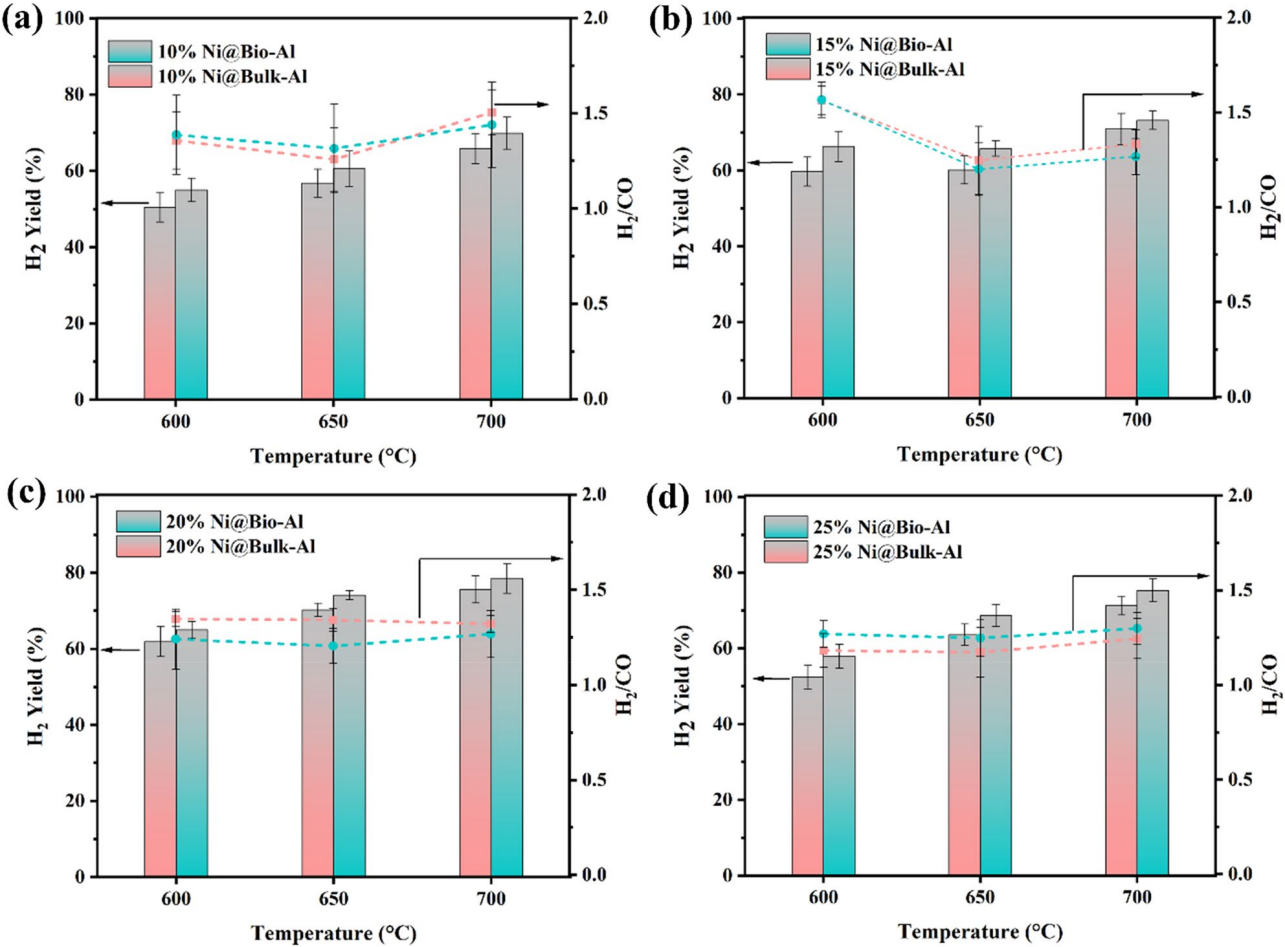
H_2_ /CO ratio and H_2_ yield for (**a**) 10% Ni, (**b**) 15% Ni, (**c**) 20% Ni, and (**d**) 25% Ni loading on bio-templated and non-templated catalysts (without Ce).

Results of H_2_ yield and H_2_/CO ratio are shown in Fig. 4a–d for different amounts of Ni loading. Regarding products, H_2_/CO molar ratio is more than unity and less than 2 in all cases due to carbon accumulation. Therefore, this catalyst is an appropriate option for applications where high amounts of hydrogen are needed. As can be seen in this figure, the highest H_2_ yield were obtained for 20Ni catalyst for both non-templated and bio-templated catalysts. It is noteworthy that the highest H_2_ yield is at 700 °C^48,51^. Regarding H_2_/CO ratio, in almost all Ni loadings, the minimum H_2_/CO ration was observed at 650 °C, either for the templated or non-templated catalyst.

By examining the effect of Ni loading amount, it is evident that increasing the Ni content from 10 to 20% enhances CH_4_ and CO_2_ conversion, and H_2_ yield. This improvement can be attributed to the availability of more active sites and larger surface areas at higher Ni loadings. However, further increasing the Ni content (25%) has a detrimental effect, resulting in decreased CH_4_ and CO_2_ conversion, and H_2_ yield. This decline could be ascribed to the agglomeration of Ni particles, leading to the loss of active sites and surface area. This agglomeration is more pronounced in higher temperatures. In summary, loading 20% Ni demonstrated the best performance, either for the bio-templated (CH_4_ and CO_2_ conversion, and H_2_ yield of 80.7%, 56.4%, and 75.8% respectively, at 700 °C), or the non-templated catalyst (CH_4_ and CO_2_ conversion of 75.4%, 52.1%, and 75.6% respectively, at 700 °C).

Figure 3 also highlights the remarkable influence of support bio-templating in achieving higher CH_4_ and CO_2_ conversion, and H_2_ yield regardless of Ni loading amount. This effect can be ascribed to multiple factors, including the more porous structure of bio-templated alumina, smaller Ni particles and their better distribution on the support in the case of bio-templated alumina compared with non-templated one, which have been confirmed by the characterization results, presented in the subsequent sections. Consequently, the bio-templating approach mitigates Ni particles’ agglomeration and is predicted to reduce coke formation and minimize catalyst deactivation. Details of excited gaseous products from the GC column are presented in Table S1.

**Figure 5:**
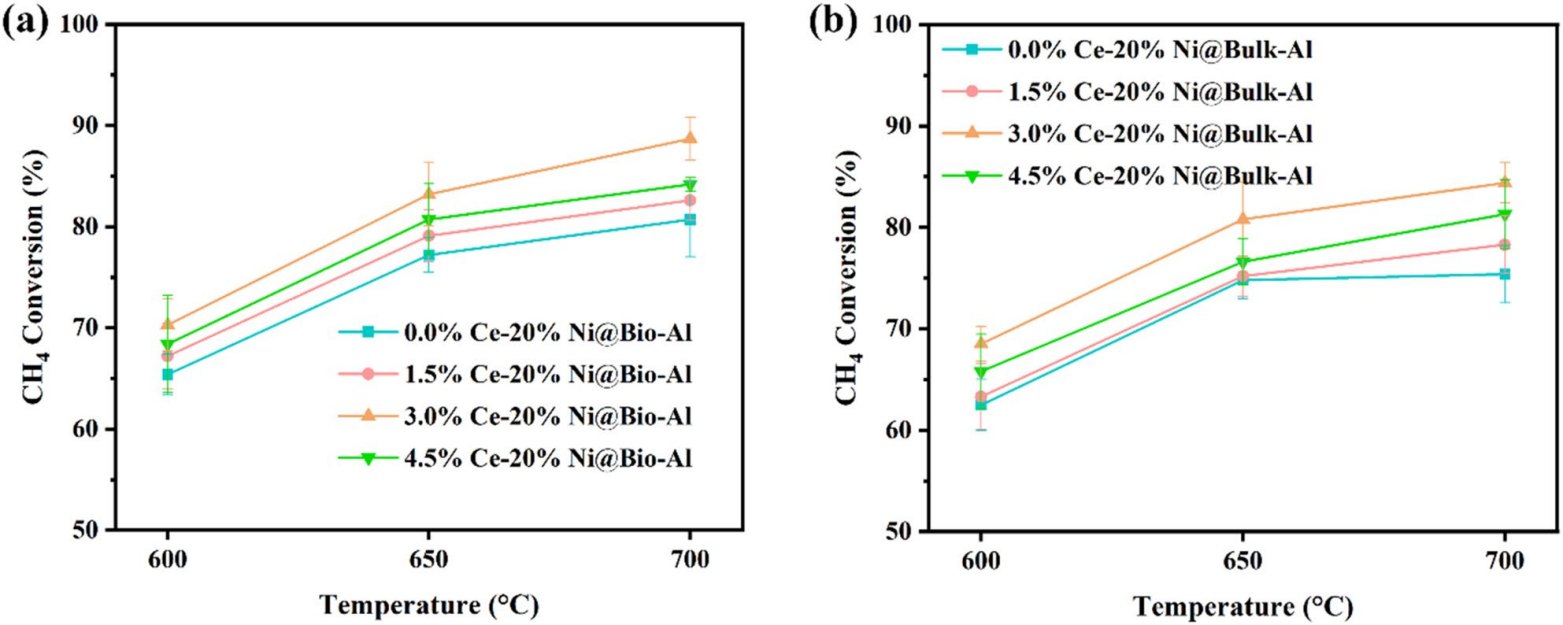
CH_4_ conversion for (**a**) bio-templated, and (**b**) non-templated Ni–Ce catalysts for 20% Ni loading and different Ce loading amounts.

To determine the effect of adding the promoter, different amounts of Ce (1.5, 3.0, and 4.5%) were loaded on both bio-templated and non-templated catalysts. CH_4_ and CO_2_ conversion and H_2_ yield were calculated for all Ni loadings amounts. The results for optimal Ni loading (20%) are presented in Figs. 5, S4, and S5. Moreover, other Ni loadings (10, 15, 25%) are shown in Figs. S3–S5. The figures demonstrate that the addition of Ce promotes higher CH_4_ conversion and H_2_ yield. This can be attributed to the activation of CO_2_ facilitated by CeO_2_ and a reduction in coke formation. When higher Ce loading is used (up to 3%), this increscent is more noticeable. At 3% Ce loading, the highest improvement in CH_4_ conversion (9.9% for bio-templated and 11.9% for non-templated catalysts) and H_2_ yield (5.7% for bio-templated and 10.6% for non-templated catalysts) were achieved at 20%Ni loading and 700 °C. However, further increasing the Ce content (4.5%) adversely affects both CH_4_ conversion and H_2_ yield, possibly due to agglomeration of Ce particles.

Similar to the previous observations, a direct relationship between temperature and catalytic performance is evident, which is due to the endothermic nature of DRM. Higher amounts of CH_4_ conversion and H_2_ yield were obtained for the bio-templated catalysts compared with the non-templated ones for all Ce loading amounts.

In summary, the optimal CH_4_ conversion and H_2_ yield of 88.7% and 85.3% were achieved by loading 20% Ni and 3% Ce on the bio-templated support and 84.4% and 83.4% on the non-templated catalysts, respectively at 700 °C. The superior performance of the bio-templated catalysts can be attributed to the presence of smaller Ni particles that are better distributed on the bio-templated support. For a better understanding, the optimized catalysts, both with and without bio-template, underwent characterizations, which the results are presented in the following section.

### Characterization of the fresh optimized catalyst

In the present study, the alumina support was synthesized in the presence of waste leaves, as shown in Fig. 1. These leaves served as a natural scaffold to improve the physical properties of catalysts. Additionally, a non-templated support was prepared for comparison purposes. To investigate the structural and morphological characteristics of both bio-templated and non-templated catalysts, various techniques were employed.

**Figure 6:**
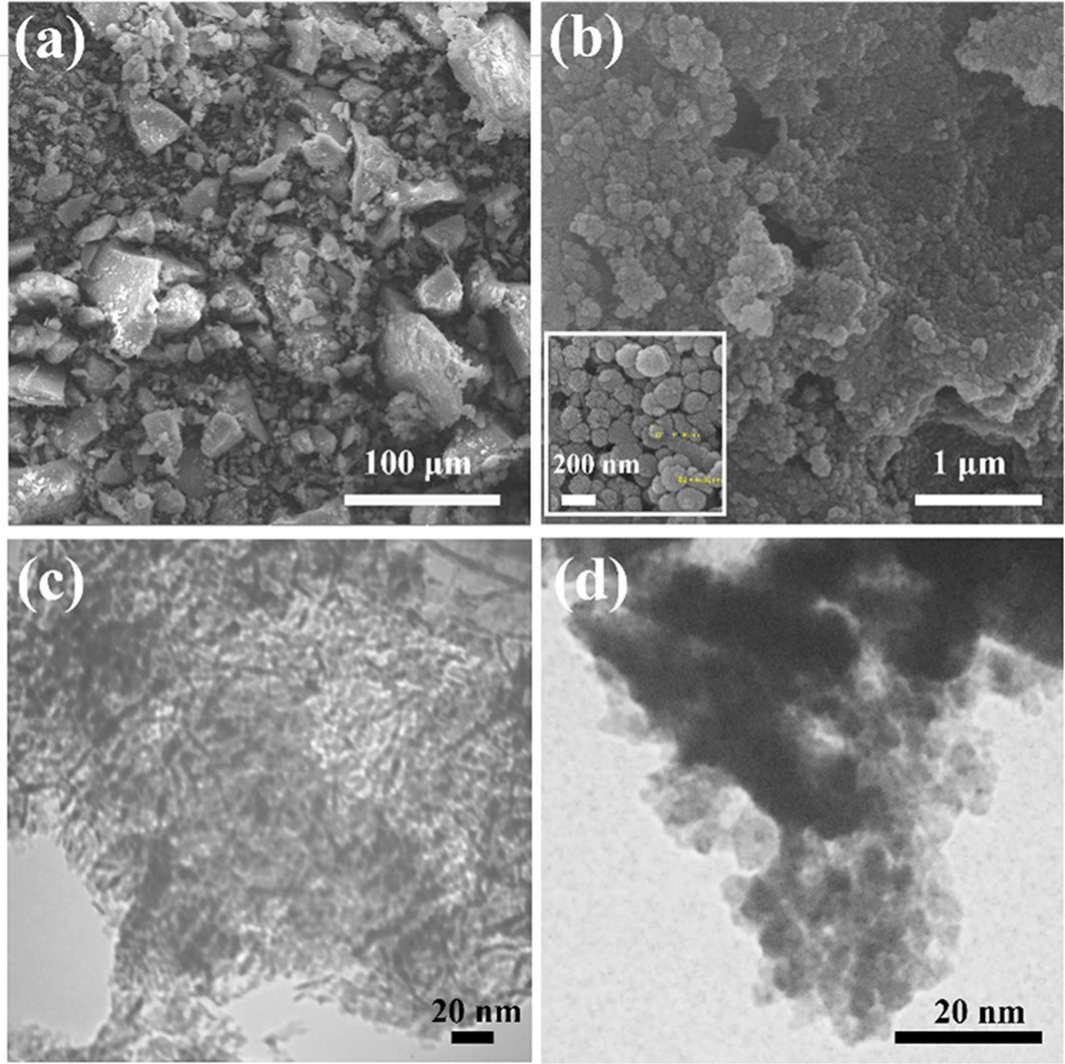
(**a**) FESEM images of non-templated, and (**b**) bio-templated alumina, (**c**) TEM image of bio-templated alumina, (**d**) TEM image of Ni–Ce@Bio-Al.

To evaluate the effect of bio-templating on catalysts’ morphology and structure, the FESEM analysis was conducted. A comparison between the FESEM images of bulk alumina and bio-templated alumina (Fig. 6a and b) shows that the non-uniform and low-porous structure of bulk alumina was remarkably affected by the bio-template and resulted in a uniform and porous structure with spherical-shape particles and size range of 44.92–91.46 nm. Moreover, the overall morphology of the leaf was successfully retained within the alumina support, which is evidence of the successful synthesis bio-templating route. This observation is further supported by the TEM image of the bio-templated alumina, as shown in Fig. 6c, which confirms the formation of an ordered and uniform mesoporous structure in the supporting material in the presence of bio-template. The TEM image in Fig. 6d presents the presence of Ni and Ce on the porous structure of alumina.

**Figure 7:**
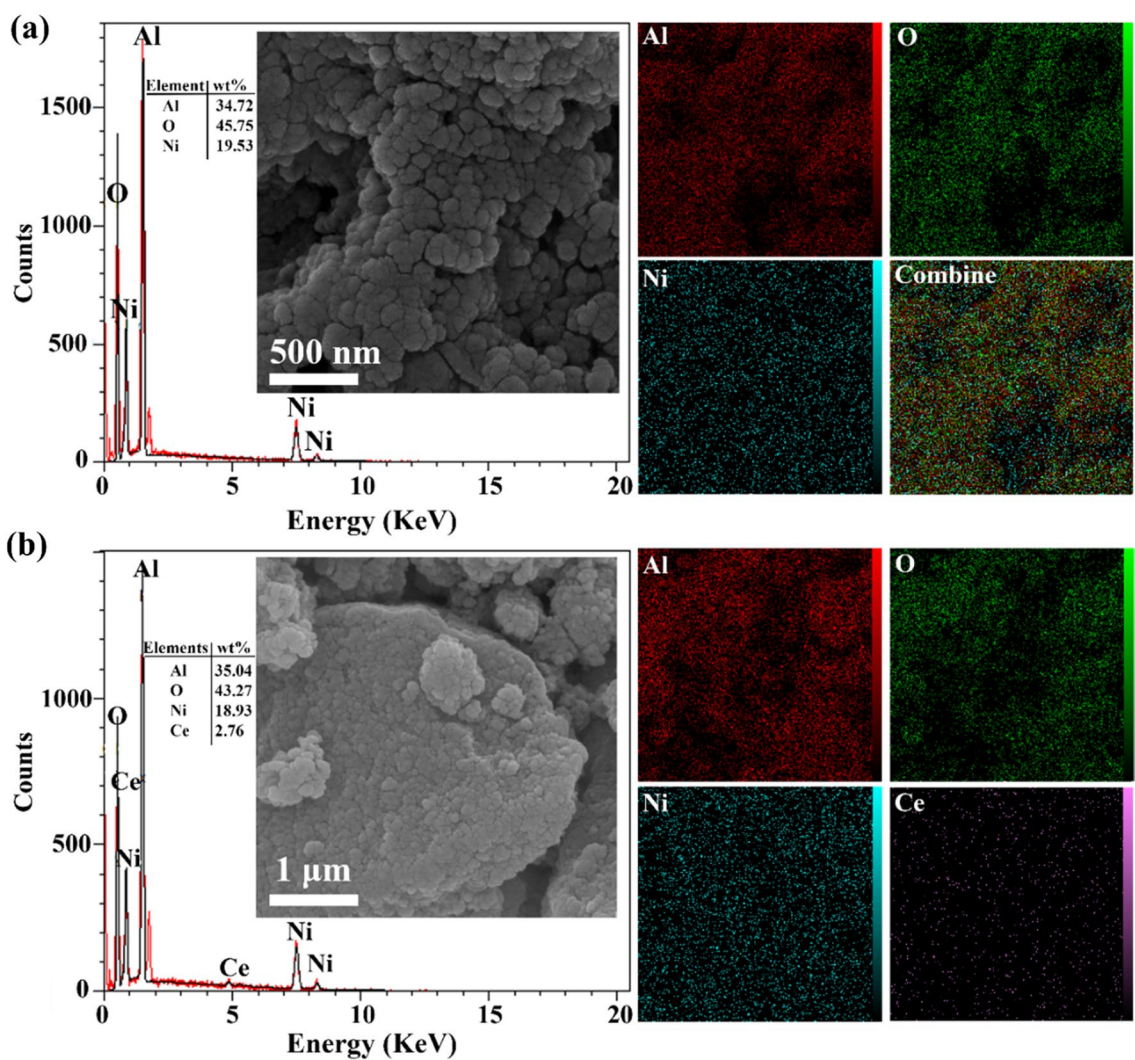
FESEM, EDX, and dot-mapping of (**a**) Ni@Bio-Al, (**b**) Ni–Ce@Bio-Al catalysts.

The templated alumina serves as an appropriate support for immobilizing Ni and Ce particles while maintaining the structure provided by the template. The agglomerated particles were observed after adding Ni to the alumina (Fig. 7a). However, after the addition 3% Ce to the 20% Ni@Bio-Al surface (Fig. 7b), well-dispersed particles with smaller sizes are observed.

To prove the presence of Ni and Ce in the prepared catalysts, EDX analysis was also conducted. The results are presented in Fig. 7a and b for 20% Ni@Bio-Al with and without 3% Ce, respectively. As expected, Al, O, Ni, (and Ce) components were detected in these samples, with a porous structure in their alumina mapping images, facilitating the effective dispersion of Ni or Ni-Ce particles on the alumina surface. The actual chemical compositions of 3%Ce-20%Ni on the templated and non-templated support using ICP analysis is presented in Table S2. The results show the presence of Ni and Ce, and highlight that bio-template did not affect the presence of Ni and Ce.

**Figure 8:**
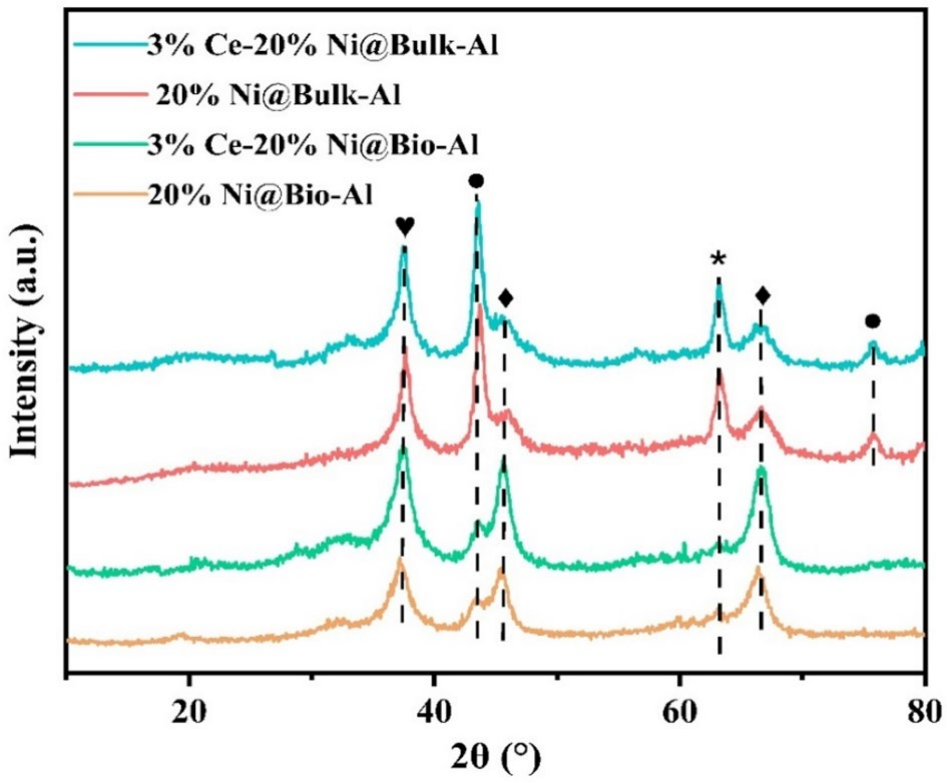
XRD spectrum of bio-templated and non-templated catalysts. The symbol (♦) represents Al_2_O_3_, (♥) NiAl_2_O_4_, (●) Ni^0^, and (*) NiO characteristic peaks.

The XRD patterns of catalysts were obtained in the 2θ range of 10–80°, in which all samples were well crystallized. Both bio-templated and non-templated catalysts showed diffraction peaks at 2θ = 45.2°, and 65.4° related to Al_2_O_3_ (Fig. 8). The presence of alumina diffraction peaks in samples after impregnation with Ni and Ce indicates that the crystal structure of alumina remained unchanged during the impregnation process. The peaks related to NiAl_2_O_4_ (at 37.5°) and Ni^0^ (at 44.5° and 75.6°) were detected in both bulk and bio-templated samples. By calcination of the samples at 650 °C, enough energy was obtained by Ni ions to overcome the surface barrier of alumina and result in spinel structure from incorporating into alumina lattice. This results in reacting NiO with Al_2_O_3_ and forming NiAl_2_O_4_. The lower intensity of Ni^0^ and NiO peaks in the bio-templated catalyst confirms the smaller size and better distribution of Ni in the templated support, which resulted in better performance of bio-template catalysts in DRM. Meanwhile, the CeO_2_ peaks could not be detected in both bio-templated and non-templated catalysts due to its low content.

**Figure 9:**
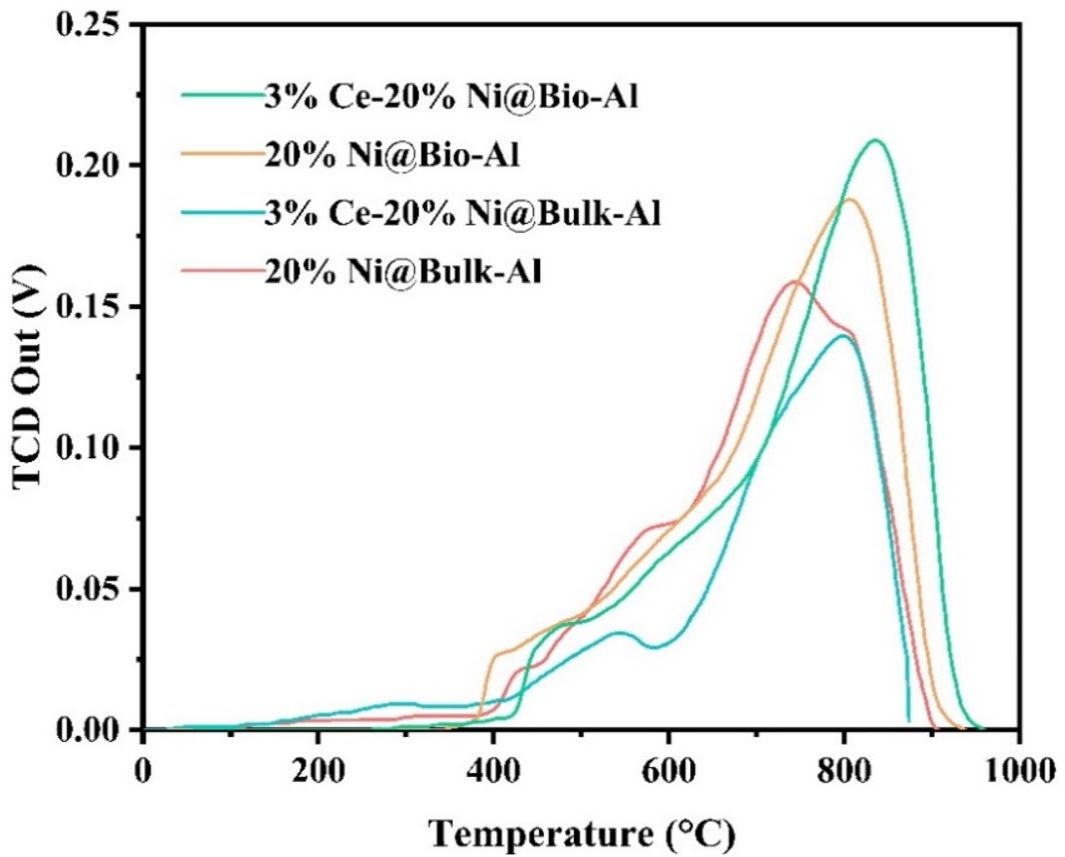
TPR profiles of bio-templated and non-templated catalysts.

The TPR analysis was carried out to determine the reducibility of nickel species on the templated and non-templated alumina surfaces and their interaction strength in the calcined catalysts^52^. As per Fig. 9, there is one main peak at more than 700 °C with a small shoulder peak at 350–550 °C for all samples. The peak in the range of 350–550 °C belongs to NiO species reduction with a low interaction with the alumina support. The peak at more than 700 °C is attributed to the Ni species strong chemical interaction with alumina support (NiAl_2_O_4_), which consists well with the XRD results. Shifting the highest peak to higher temperatures for bio-templated catalysts presents a stronger interaction between Ni and Al. Therefore, the Ni particles are more properly distributed by bio-templating, which is consistent with XRD results Compared with the non-promoted catalysts, the promoted catalysts showed a higher reduction temperature, which indicates much stronger metal-support interaction which will result in a higher stability of the catalyst. Although adding Ce did not change the place of peaks significantly, more hydrogen is consumed in the promoted catalyst, which shows a higher oxygen storage capacity of 3% Ce–20% Ni@Bio-Al catalyst than 20% Ni@Bio-Al, and thus its lower tendency for coke deposition during the DRM reaction. This indicates that bio-templating promoted the formation of Ni species that had a strong interaction with the support, which is consistent with the XRD results.

**Figure 10:**
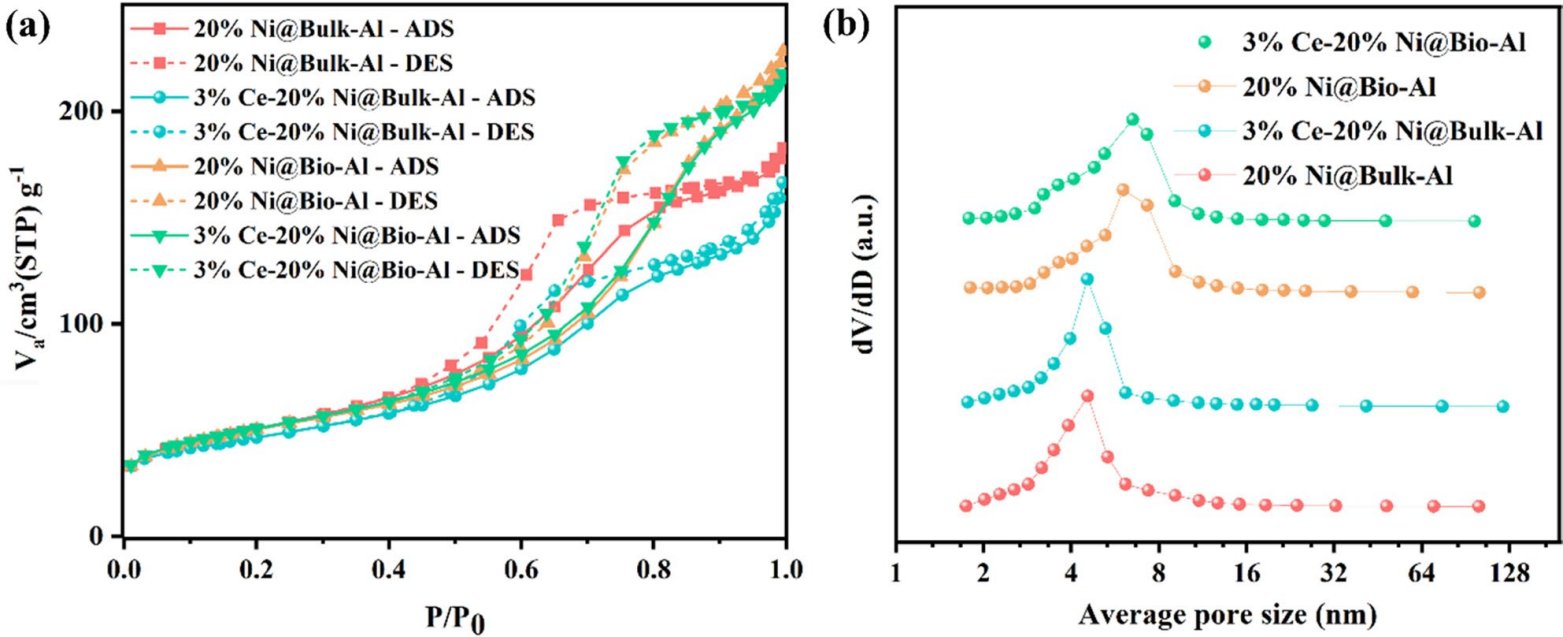
(**a**) N_2_ adsorption/desorption isotherms and (**b**) pore size distribution of 3% Ce and 20% Ni on bio-templated and non-templated catalysts.

The textural properties of catalysts with and without bio-template were determined via the N_2_ adsorption–desorption analysis, which results are summarized in Table 1. The adsorption–desorption isotherms and the pore size distributions are also shown in Fig. 10a and b, respectively. For all samples, the isotherm presented a typical type IV curve with H3 shaped hysteresis loop. The hysteresis loops for all samples started at about 0.4, which was characteristic of mesoporous material, as was confirmed by the FESEM and TEM analysis. The uniformity of mesoporous structure is proven by the sharpness of the capillary condensation stage. Figure 10b illustrates a narrow pore size distribution in approximately equal center points of 4.00nm for 20%Ni@Bio-Al and 3% Ce–20% Ni @Bio-Al samples. The sharper peak in Bio-Al supporting material indicates a higher number of pores in this confined range and a blockage of some pores by Ni and/or Ni–Ce particles, as indicated by the adsorption and desorption diagrams. As per Table 1, the specific BET surface areas and pore volumes of each sample are different. The bio-templated samples showed higher textural properties compared with the non-templated samples, which shows the synergic effect of bio-template in increasing the surface area of catalyst. After impregnating Ni, the surface area of templated catalyst decreased from 214.01 m^2^/g of pure Al_2_O_3_ to 173.23 m^2^/g^19,53,54^. The BET surface area of the promoted sample did not change significantly against the un-promoted one, which can be correlated to the enhancement effect of CeO_2_ particles on the dispersion of NiO active sites that caused smaller particles and thus lower pore blockage of the supporting material. A slight reduction in surface area can be attributed to the deposition of Ce inside the catalyst. Therefore, the pore volume and pore size of promoted catalysts become somewhat smaller than un-promoted ones as still small pores of alumina support are not covered by active sites.

**Table 1:** N_2_ adsorption/desorption results for bio-templated and non-templated catalysts.

Sample name	BET surface area (m^2^/gr)	Pore diameter (Using adsorption branch) (nm)	Pore volume (Based on BJH method) (cm^3^/gr)
Bio-Al	214.01	3.89	0.48
20% Ni@Bio-Al	173.23	4.03	0.44
3% Ce−20% Ni @Bio-Al	171.87	4.03	0.45
Bulk-Al	172.43	5.43	0.32
20% Ni@Bulk-Al	124.64	6.01	0.33
3% Ce−20% Ni @Bulk-Al	120.07	6.02	0.33

### Stability test during long-term reaction

**Figure 11:**
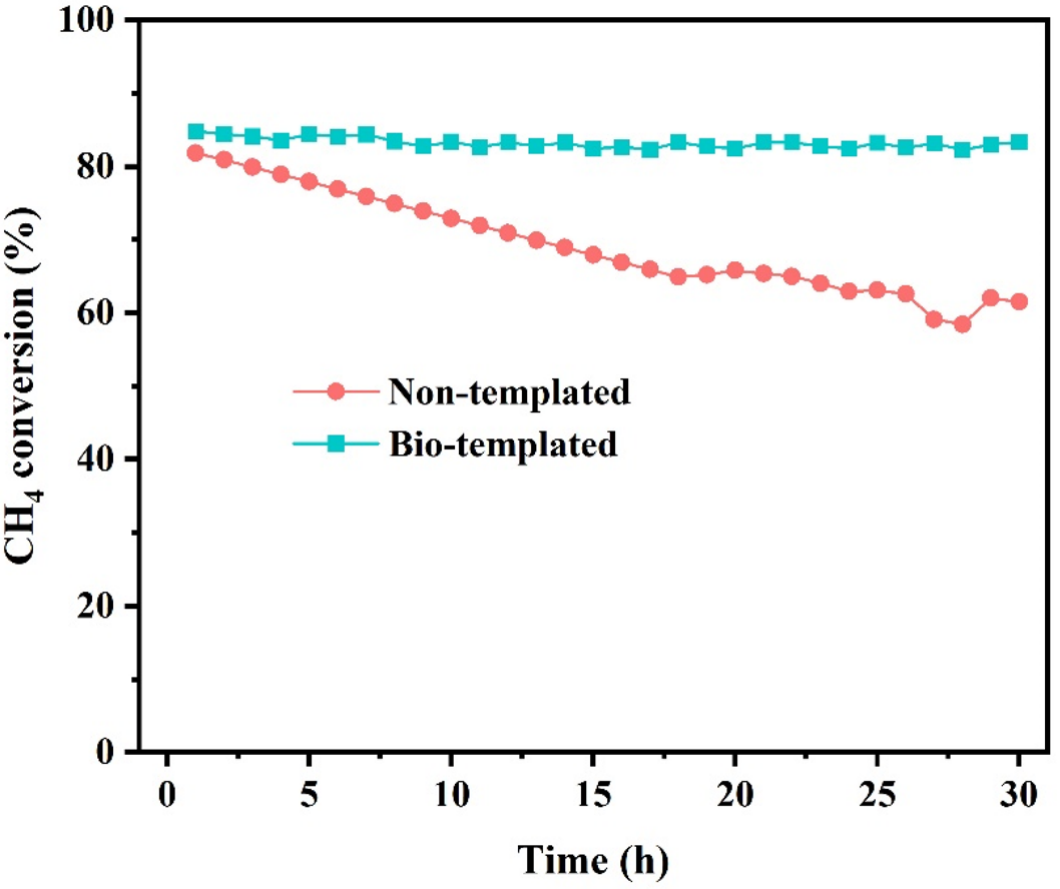
Time on stream reaction for bio-templated and non-templated catalysts.

The time-on stream reaction tests were carried out for 30 h at 650 °C using the optimized 3% Ce- 20% Ni on bio-templated and non-templated catalysts to evaluate their stability and coke resistance. Figure 11 shows the results for CH_4_ conversion. The initial CH_4_ conversions were 84.8% and 81.8% for the bio-templated and non-templated catalysts, respectively. The methane conversion of bio-templated catalyst remained almost stable and only a 1.76% reduction resulted after 30 h reaction, showing its good stability. It is while, the non-templated catalyst experienced a 24.81% loss in CH_4_ conversion after 30h, indicating its less stability compared to the bio-templated catalyst. These results present less coke formation and sintering for the bio-templated catalyst, which is consistent well with what expected from the TPR and XRD results of fresh bio-templated and non-templated catalysts (Figs. 8 and 9) and stability is more in case of bio-templated catalyst as expected. Due to the higher stability of the bio-templated catalysts, the characterization analysis was done for this catalyst after 30 h long-time reaction to determine the factors participating in the catalyst deactivation, which are presented in the following.

**Figure 12:**
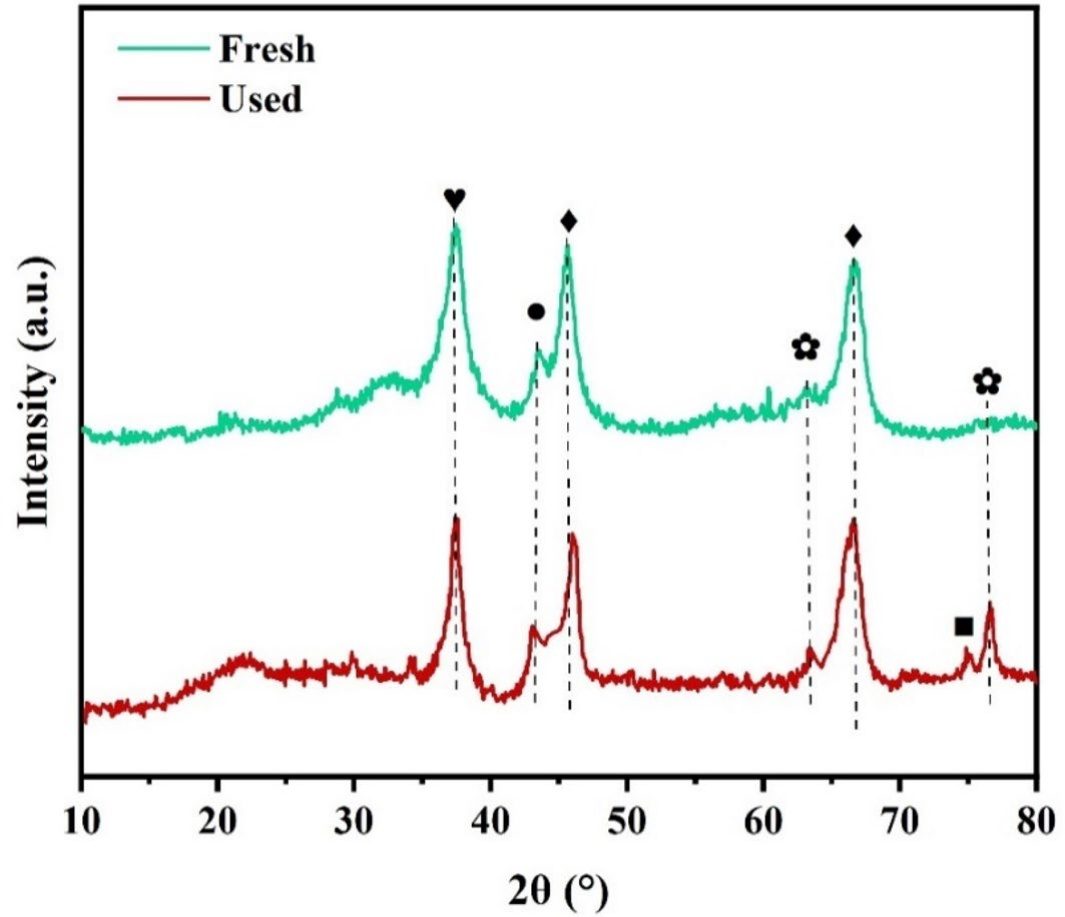
XRD patterns of bio-templated and non-templated catalysts before and after DRM reaction. Symbol (♦) represents Al_2_O_3_, (♥) NiAl_2_O_4_ (●) Ni^0^, (■) C, (✿) NiO.

The XRD patterns of the bio-templated catalyst before and after the reaction are depicted in Fig. 12. In the pristine catalyst, the presence of Al_2_O_3_, NiO, Ni^0^, and NiAl_2_O_4_ phases was confirmed. However, in the used catalyst, additional carbon peaks emerged, indicating the formation of coke. Furthermore, the intensity of NiO and Ni^0^ peaks was higher, suggesting a larger particle size due to sintering. These results show that despite the bio-templated catalyst experiencing slight sintering and coke formation during the 30 h DRM reaction, it has good stability, which is in line with the FESEM, TGA, and BET results.

**Figure 13:**
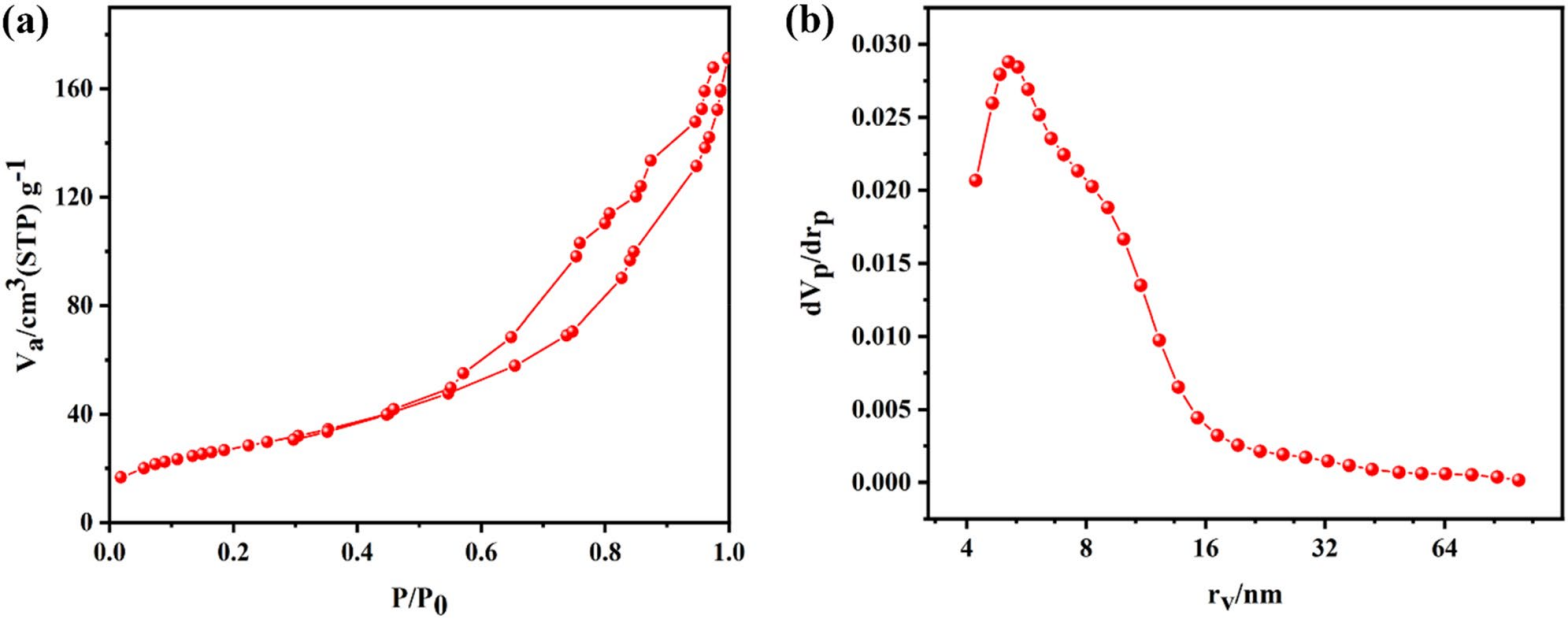
N_2_ adsorption/desorption isotherms and pore size distribution of 3% Ce–20% Ni@Bio-Al sample before and after DRM reaction.

The N_2_ adsorption/desorption isotherm of 3% Ce-20% Ni @Bio-Al after 30 h of the DRM reaction is illustrated in Fig. 13a. According to this figure, the type of isotherm is same as its fresh state (IV type with an H3 hysteresis loop), showing that the bio-template catalyst’s structure did not change after 30h reaction. However, its hysteresis loop becomes closer after the reaction, possibly due to the blockage of small pores by deposited coke or the sintering of the catalyst at high temperatures. As a result, its BET surface area had reduced by 7.85% and its pore size (Fig. 13b) and pore volume were increased slightly due to coke formation, which was also confirmed by the XRD results (Table 2).

**Table 2:** N_2_ adsorption/desorption results of 3% Ce–20% Ni@Bio-Al sample after DRM reaction.

Sample name	BET surface area (m^2^/gr)	Pore diameter (nm)	Pore volume (cm^3^/gr)
3% Ce−20% Ni **@**Bio-Al	150.07	5.1	0.48

**Figure 14:**
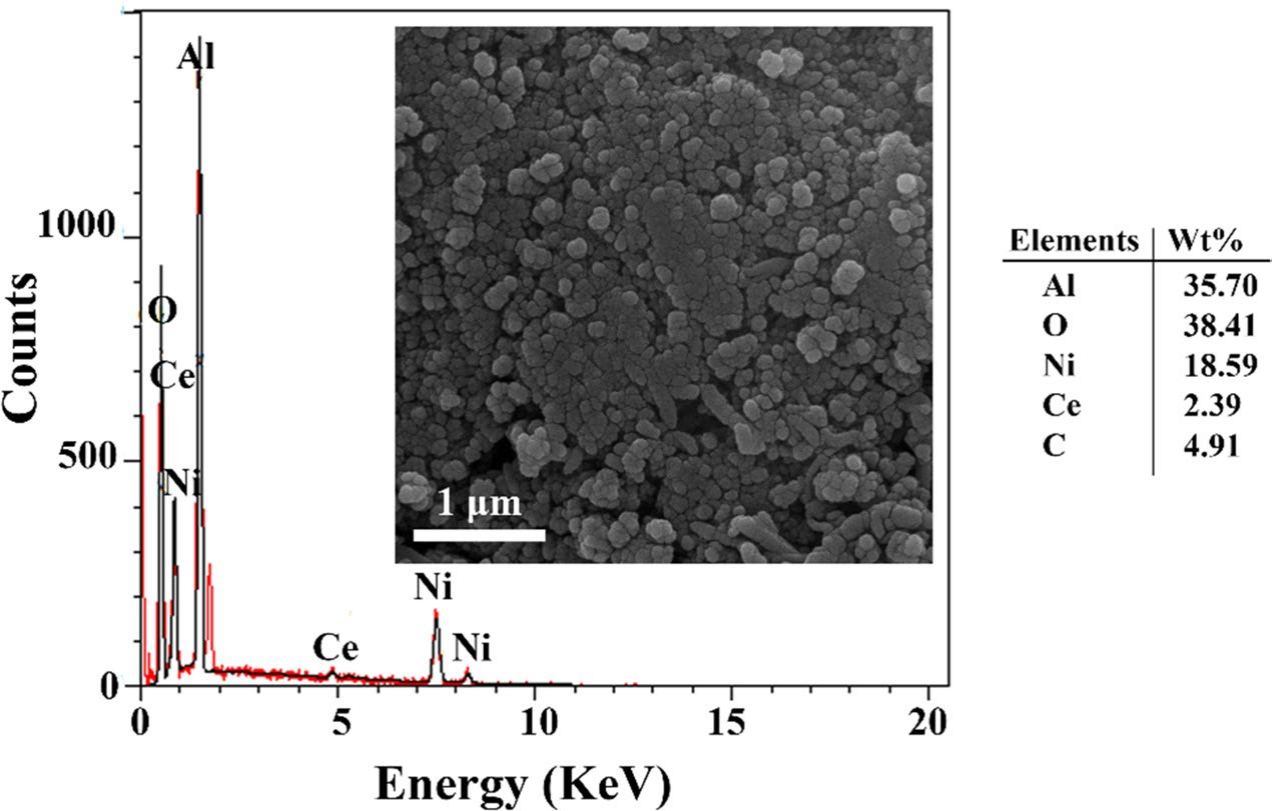
FESEM and EDX of 3% Ce-20% Ni @Bio-Al catalyst after DRM reaction.

The FESEM and EDX results of 3% Ce- 20% Ni@Bio-Al are shown in Fig. 14 to determine the amount of coke formation. According to the results of the elemental composition the elements of Al, Ni, Ce, C, and O have been detected in this catalyst with a low carbon content of 4.91 wt% after 30 h DRM time-on-stream. This observation is in line with the findings of the FESEM image, in which dark areas that present coke deposition on the catalyst surface are not observed. The comparison between FESEM images of 3% Ce-20% Ni @Bio-Al before and after the high-temperature DRM reaction indicates that some Ni particles are sintered and stuck together after the reaction.

**Figure 15:**
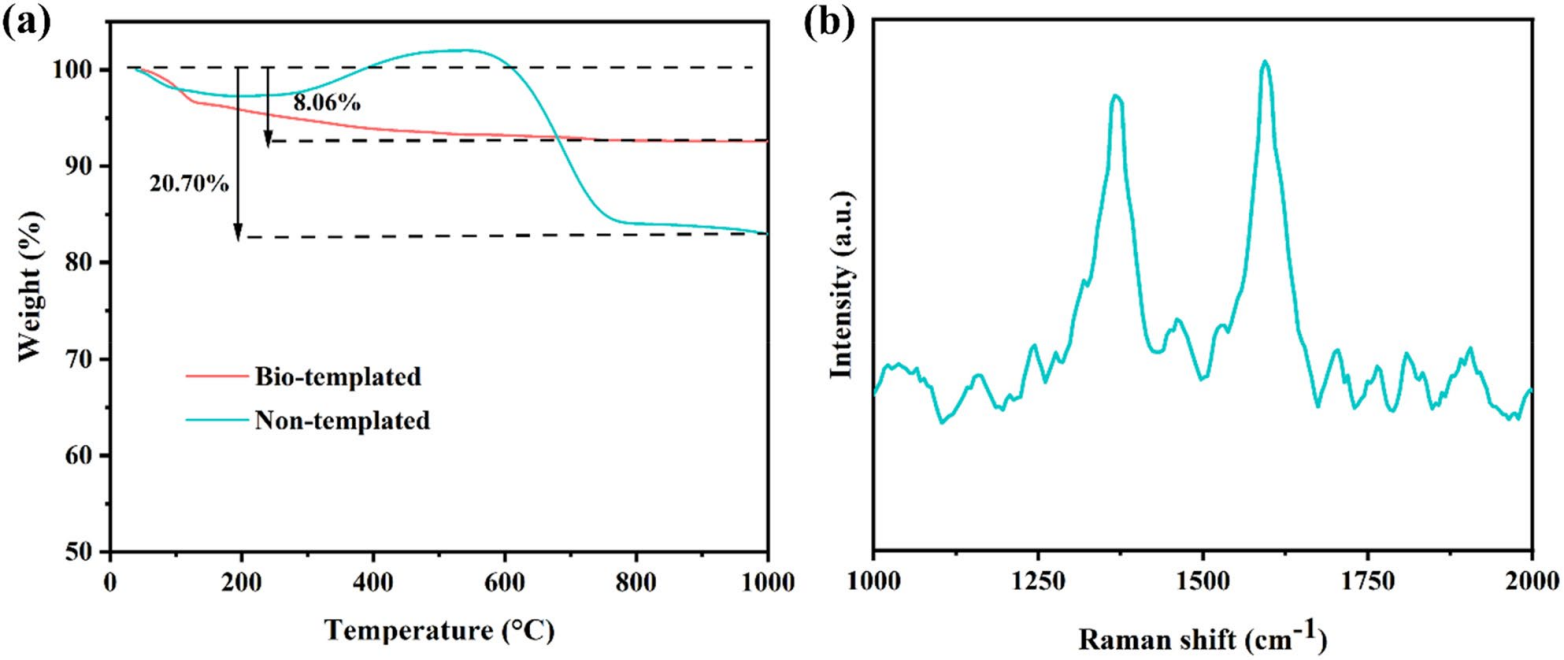
(**a**) TGA profile of spent bio-templated and non-templated catalysts, (**b**) Raman spectra of spent non-template catalyst.

TGA analysis was used to evaluate the thermal stability of the catalyst and determine the amount of carbon deposited on the catalyst’s surface after 30 h DRM reaction at 650 °C. Given that, the fresh catalyst was heated to 1000 °C under Ar media with 20 °C/min heating ramp, while the used catalyst was heated to this temperature using air molecules to burn the coke deposited on its surface. As presented in Fig. 15, the bio-templated catalyst showed a sharp reduction in its weight percentage before 100 °C, which is caused by the desorption of H_2_O and other adsorbed reactants^55^. For the case of the non-templated catalyst, the weight of the used catalyst initially decreased until 200 °C (Fig. 15a) due to the desorption of adsorbed water molecules. Following this reduction, an increment was observed in the weight of the non-templated catalyst between 200 °C and 600 °C due to re-oxidation of the Ni active sites under oxidative atmosphere. In this case TGA profile is not reliable to determine the type of coke and the Raman analysis was further done to analyze the type of carbon in non-templated catalyst. From the Raman spectra in Fig. 15b two main peaks at 1361.098 cm^−1^ (disordered sp^2^ hybridized carbons named D band) and 1588.67 cm^−1^ (sp^2^ hybridized graphite-like carbons named G band) are observed, which are respectively correspond to amorphous and graphitic carbon^12–15,56,57^. The sharp loss of weight after 600 °C is the result of oxidation of deposited carbon on 3%Ce- 20%Ni@Bulk-Al after 30h DRM reaction at 650 °C. It was assumed that there was no significant deposited carbon oxidation below 600 °C. The bio-templated catalyst’s weight decreased slightly by only about 8.06% until the end of the analysis, representing the bio-templated catalyst’s good thermal stability. It is while the total weight loss was 20.70% for the non-templated catalyst. Typically, three types of carbon can be formed and detected using TGA analysis; amorphous carbon at about 320 °C, filament carbon in the range of 320–520 °C, and graphitic carbon at temperatures more than 650 °C. Considering this, the detected deposited carbon on bio-templated catalysts is attributed to amorphous and filament carbon.

### Thermodynamic analysis of DRM

**Figure 16:**
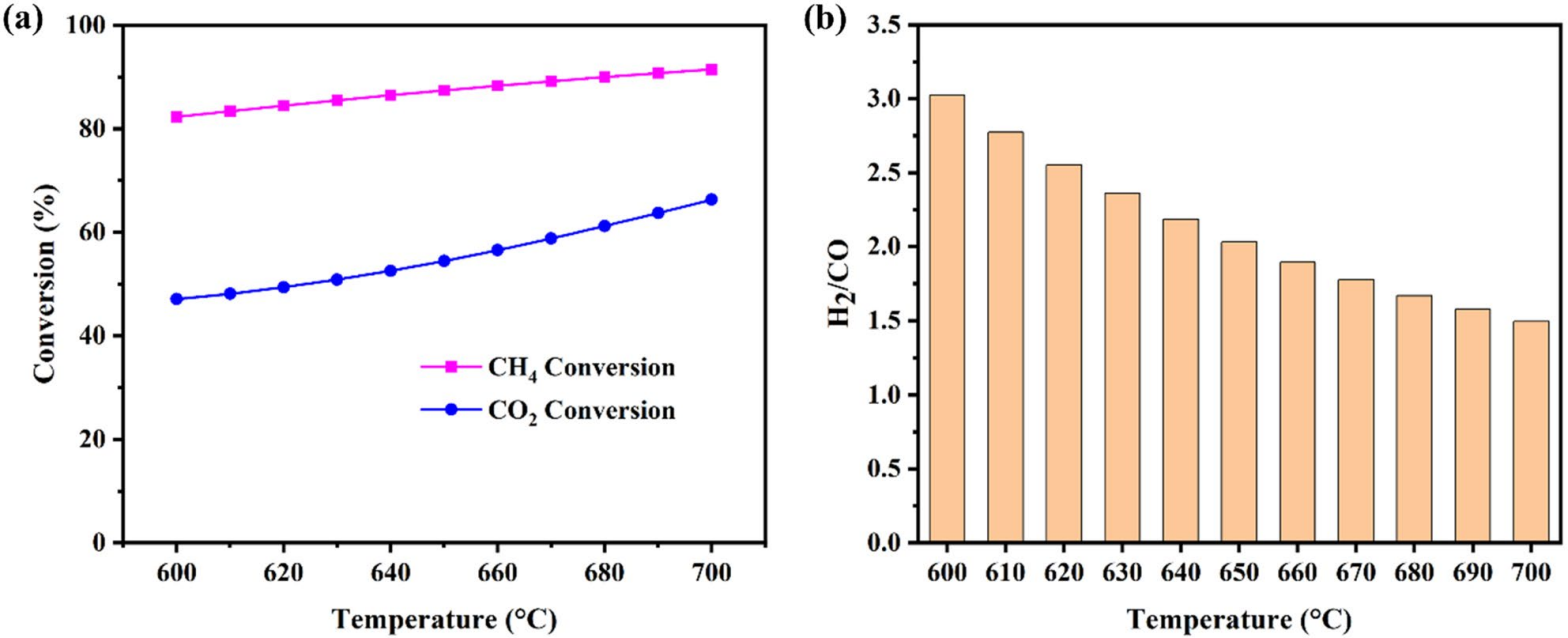
Equilibrium (**a**) CH_4_ and CO_2_ conversion, (**b**) H_2_/CO ratio as a function of temperature (CO_2_/CH_4_ = 1, P = 1 atm).

The results of thermodynamic equilibrium studies for CH_4_, CO_2_ conversion, and H_2_/CO ratio in products at the temperature range of 600–700 °C is shown in Fig. 16a and b. It is noteworthy that the feed ratio was CH_4_:CO_2_ = 1:1 and pressure was 1 atm.

It is evident that temperature has a significant effect on CH_4_ and CO_2_ conversions. The CH_4_ and CO_2_ conversions are small at lower temperatures (600 °C), while they are enhanced by increasing temperature to 700 °C. For the strong endothermic reaction of DRM, the equilibrium conversion of CH_4_ and CO_2_ increases dramatically with increasing reaction temperature. Thus, high conversion is favored at high temperatures. The moderate endothermic reactions, methane decomposition, and the reverse water–gas shift reaction, also increase with temperature. The two carbon deposition reactions, the Boudouard reaction and the reverse carbon gasification reaction, are exothermic and thermodynamically unfavorable at high temperatures. Therefore, high reaction temperatures (i.e., 700 °C and above) are more favorable to increasing the equilibrium conversion of the main reaction than that of the side reactions.

### Comparing results in present work with the similar studies

For the sake of fair comparison, the performance of the best catalyst evaluated in the present study (20%Ni-3%Ce on bio-templated alumina at 700 °C) with similar works in previous studies in the DRM reaction, Table 3 is presented. According to Table 3, CH_4_ conversion varies between 54.5% and 94%, using different promoted or unpromoted Ni–Al_2_O_3_ catalysts and various operational conditions. A comparison between the achieved CH_4_ conversion in this work with similar studies, considering the reaction condition, shows higher activity of our bio-templated Ni–Al_2_O_3_ catalyst.

**Table 3:** A comparison of the CH_4_ conversion for similar studies.

Support	Active phase/promoter	Reaction condition	CH_4_ conversion	Ref
Al_2_O_3_	10% Ni 0–0.7% B	CH_4_:CO_2_ = 1:1 T = 700 °C GHSV = 30,000 ml/g h	54.5	^19^
Al_2_O_3_	5%Ni 3%Mg	CH_4_:CO_2_ = 1:1 T = 700 °C GHSV = 12,000 ml/g h	74	^38^
Al_2_O_3_	20%Ni 2%Yb	CH_4_:CO_2_ = 1:1 T = 700 °C GHSV = 52,000 ml/g h	80	^35^
Al_2_O_3_	Ni 8% Ce	CH_4_:CO_2_		^32^
Al_2_O_3_	10%Ni 6% La	CH_4_:CO_2_ = 1:1 T = 800 °C GHSV = 6000 ml/g h	93	^36^
Al_2_O_3_	10%Ni 0.7%Fe	CH_4_:CO_2_ = 1:1 T = 700 °C GHSV = 24,000 ml/g h		^58^
Al_2_O_3_	25%Ni	CH_4_:CO_2_ = 1:1 T = 700 °C GHSV = 12,000 ml/g h	70%	^59^
Al_2_O_3_	Ni	CH_4_:CO_2_:N_2_ = 2:2:1 T = 700 °C GHSV = 37,600 ml/g h	88%	^60^
Al_2_O_3_	15% Ni	CH_4_:CO_2_:N_2_ = 1:1:4 T = 700 °C GHSV = 72,000 ml/g h	76%	^61^
Al_2_O_3_ Bio-templated	20% Ni 3% Ce	CH_4_:CO_2_ = 1:1 T = 700 °C GHSV = 12,000 ml/ g h	88.7%	This work

## Conclusion

In the present study, the waste leaves of Sycamore were utilized as the scaffold to prepare alumina for catalyst support, with the subsequent co-impregnating Ni and Ce on the bio-templated alumina. Comparing the activity of a bio-templated catalyst with a non-templated one in the same condition proved that the bio-templated catalyst has higher efficiency and excellent stability in DRM reaction. The enhanced catalytic performance can be attributed to higher surface area, improved Ni dispersion on support, and stronger Ni–Al interaction in bio-templated catalyst compared to the non-templated one. The optimal catalytic performance was achieved with 20% Ni and 3% Ce loading at 700 °C for both catalyst types. The successful bio-template synthesis approach was evident from the FESEM images, which reveals a uniform and highly porous structure. XRD, TPR, and N_2_ adsorption/desorption analyzes confirmed strong interaction between nickel and alumina and enhanced surface area in templated catalyst. The templated catalyst showed enhanced stability, reduced catalyst deactivation by coke formation and sintering during 30 h DRM.

### Plant material

The waste leaves of Sycamore that fell in autumn were collected as the bio-template under the Environmental Organization of Shiraz University by considering Institute of Standards & Industrial Research of Iran (http://www.isiri.com).

### Supplementary Information

Supplementary Information 1.

Supplementary Information 2.

## Data Availability

All data generated or analysed during this study are included in this published article.

## References

[1] Sheng KF, Cui K (2023). Highly dispersed Ni nanoparticles supported by porous Al_2_O_3_ rods for catalytic dry reforming of methane. New J. Chem..

[2] Saidur R, Rahim NA, Islam MR, Solangi KH (2011). Environmental impact of wind energy. Renew. Sustain. Energy Rev..

[3] Zhu Q, Zhou H, Wang L, Wang L, Wang C, Wang H, Fang W, He M, Wu Q, Xiao F-S (2022). Enhanced CO_2_ utilization in dry reforming of methane achieved through nickel-mediated hydrogen spillover in zeolite crystals. Nat. Catal..

[4] Zain MM, Mohamed AR (2018). An overview on conversion technologies to produce value added products from CH_4_ and CO_2_ as major biogas constituents. Renew. Sustain. Energy Rev..

[5] Hamzehlouia S, Jaffer SA, Chaouki J (2018). Microwave heating-assisted catalytic dry reforming of methane to syngas. Sci. Rep..

[6] Kwon Y, Eichler JE, Floto ME, Mullins CB (2023). The complementary relationship between Ru/Al_2_O_3_ and Ni/Al_2_O_3_ catalyst for dry reforming of methane. Chem. Eng. Res. Des..

[7] Aryafard E, Farsi M, Alipoor AR, Rahimpour MR (2023). The effect of CO_2_ feeding on syngas quality in autothermal reforming of methane over Ni-Al_2_O_3_ catalyst: possibility of CO_2_ conversion to hydrogen. React. Kinet. Mech. Catal..

[8] Lubitz W, Tumas W (2007). Hydrogen: An overview. Chem. Rev..

[9] Paksoy AI, Caglayan BS, Aksoylu AE (2015). A study on characterization and methane dry reforming performance of Co–Ce/ZrO_2_ catalyst. Appl. Catal. B Environ..

[10] Bradford MCJ, Vannice MA (1999). CO_2_ reforming of CH_4_. Catal. Rev.-Sci. Eng..

[11] Yentekakis IV, Goula G (2017). Biogas management: Advanced utilization for production of renewable energy and added-value chemicals. Front. Environ. Sci..

[12] le Saché E, Pastor-Pérez L, Watson D, Sepúlveda-Escribano A, Reina TR (2018). Ni stabilised on inorganic complex structures: Superior catalysts for chemical CO_2_ recycling via dry reforming of methane. Appl. Catal. B Environ..

[13] Mohandessi M, Kiani MR, Yousefi S, Rahimpour MR (2023). Tuning the basicity of the Ni@MCM-41 catalyst via alkaline earth metal oxide promoters for CO_2_ reforming of CH_4_. React. Chem. Eng..

[14] Rockström J (2017). A roadmap for rapid decarbonization. Science.

[15] Hu YH, Ruckenstein E (2020). Dry reforming of methane by stable Ni–Mo nanocatalysts on single-crystalline MgO. Science.

[16] Ha H (2017). Design of reduction process of SnO_2_ by CH_4_ for efficient Sn recovery. Sci. Rep..

[17] Mohandessi M, Tavakolian M, Rahimpour MR (2023). Cadmium as a robust and novel promoter over Ni@γ–Al_2_O_3_ catalysts in dry methane reforming. ACS Appl. Energy Mater..

[18] Kurlov A (2020). Exploiting two-dimensional morphology of molybdenum oxycarbide to enable efficient catalytic dry reforming of methane. Nat. Commun..

[19] Fouskas A, Kollia M, Kambolis A, Papadopoulou C, Matralis H (2014). Boron-modified Ni/Al_2_O_3_ catalysts for reduced carbon deposition during dry reforming of methane. Appl. Catal. A Gen..

[20] Jang WJ (2016). Combined steam and carbon dioxide reforming of methane and side reactions: Thermodynamic equilibrium analysis and experimental application. Appl. Energy.

[21] Jang WJ, Shim JO, Kim HM, Yoo SY, Roh HS (2019). A review on dry reforming of methane in aspect of catalytic properties. Catal. Today.

[22] Pt M (2023). ScienceDirect Dry reforming of methane for syngas production over noble metals modified M–Ni @ S-1 catalysts. Int. J. Hydrogen Energy.

[23] Zhang ZY, Li T, Yao JL, Xie T, Xiao Q (2023). Mechanism and kinetic characteristics of photo-thermal dry reforming of methane on Pt/mesoporous-TiO_2_ catalyst. Mol. Catal..

[24] Al-Fatesh, A. S., Chava, R., Appari, S., Almutairi, G., Fakeeha, A. H., Ibrahim, A. A., Alromaeh, A. I., Abasaeed, A. E. & Abu-Dahrieh, J. K. Effect of Ni–Co addition on Pd promoted Al_2_O_3_ catalysts for dry reforming of methane. *SSRN 4505759* **8**, 324–350 (2023).

[25] Mao Y (2023). Coke-resistance over Rh–Ni bimetallic catalyst for low temperature dry reforming of methane. Int. J. Hydrogen Energy.

[26] Akri M (2019). Atomically dispersed nickel as coke-resistant active sites for methane dry reforming. Nat. Commun..

[27] Al-Fatesh AS (2020). Promotional effect of magnesium oxide for a stable nickel-based catalyst in dry reforming of methane. Sci. Rep..

[28] Zhu YA, Chen D, Zhou XG, Yuan WK (2009). DFT studies of dry reforming of methane on Ni catalyst. Catal. Today.

[29] Chen D, Lødeng R, Anundskås A, Olsvik O, Holmen A (2001). Deactivation during carbon dioxide reforming of methane over Ni catalyst: Microkinetic analysis. Chem. Eng. Sci..

[30] Yoo E (2023). Effects of operating parameters and feed gas compositions on the dry reforming of methane over the Ni/Al_2_O_3_ catalyst. Catalysts.

[31] Saelee T (2021). Experimental and computational investigation on underlying factors promoting high coke resistance in NiCo bimetallic catalysts during dry reforming of methane. Sci. Rep..

[32] Laosiripojana N, Sutthisripok W, Assabumrungrat S (2005). Synthesis gas production from dry reforming of methane over CeO_2_ doped Ni/Al_2_O_3_: Influence of the doping ceria on the resistance toward carbon formation. Chem. Eng. J..

[33] Pechimuthu NA, Pant KK, Dhingra SC (2007). Deactivation studies over Ni–K/CeO_2_–Al_2_O_3_ catalyst for dry reforming of methane. Ind. Eng. Chem. Res..

[34] Jin B, Wang K, Yu H, He X, Liang X (2023). Engineering oxygen vacancy-rich CeO_x_ overcoating onto Ni/Al_2_O_3_ by atomic layer deposition for bi-reforming of methane. Chem. Eng. J..

[35] Amin MH, Mantri K, Newnham J, Tardio J, Bhargava SK (2012). Highly stable ytterbium promoted Ni/γ–Al_2_O_3_ catalysts for carbon dioxide reforming of methane. Appl. Catal. B Environ..

[36] Xu J, Zhou W, Wang J, Li Z, Ma J (2009). Characterization and analysis of carbon deposited during the dry reforming of methane over Ni/La_2_O_3_/Al_2_O_3_ catalysts. Cuihua Xuebao/Chinese J. Catal..

[37] Alipour Z, Rezaei M, Meshkani F (2014). Effect of Ni loadings on the activity and coke formation of MgO-modified Ni/Al_2_O_3_ nanocatalyst in dry reforming of methane. J. Energy Chem..

[38] Alipour Z, Rezaei M, Meshkani F (2014). Effects of support modifiers on the catalytic performance of Ni/Al_2_O_3_ catalyst in CO_2_ reforming of methane. Fuel.

[39] Roostaei T (2023). Recent advances and progress in biotemplate catalysts for electrochemical energy storage and conversion. Adv. Colloid Interface Sci..

[40] Jiang X, Li W, Guo Y, Wang L, Li Q, Li Q (2019). Progress on bio-templated synthesis of metal oxides and their catalytic applications. Chem. Ind. Eng. Prog..

[41] Vijayaraghavan K, Nalini SPK (2010). Biotemplates in the green synthesis of silver nanoparticles. Biotechnol. J..

[42] Knez M (2003). Biotemplate synthesis of 3 nm nickel and cobalt nanowires. Nano Lett..

[43] Zhou H, Fan T, Zhang D (2007). Hydrothermal synthesis of ZnO hollow spheres using spherobacterium as biotemplates. Microporous Mesoporous Mater..

[44] Wang N (2013). Facile route for synthesizing ordered mesoporous Ni–Ce–Al oxide materials and their catalytic performance for methane dry reforming to hydrogen and syngas. ACS Catal..

[45] Ocsachoque M, Pompeo F, Gonzalez G (2011). Rh–Ni/CeO_2_–Al_2_O_3_ catalysts for methane dry reforming. Catal. Today.

[46] Anh AN (2023). Insight into the role of material basicity in the coke formation and performance of Ni/Al_2_O_3_ catalyst for the simulated-biogas dry reforming. J. Energy Inst..

[47] Saconsint S (2023). Development of Ni–Mo carbide catalyst for production of syngas and CNTs by dry reforming of biogas. Sci. Rep..

[48] Adamu S, Bawah AR, Muraza O, Malaibari Z, Hossain MM (2020). Effects of metal support interaction on dry reforming of methane over Ni/Ce–Al_2_O_3_ catalysts. Can. J. Chem. Eng..

[49] Shah M, Das S, Nayak AK, Mondal P, Bordoloi A (2018). Smart designing of metal-support interface for imperishable dry reforming catalyst. Appl. Catal. A Gen..

[50] Teo SH, Yap DKY, Mansir N, Islam A, Taufiq-Yap YH (2019). Facile recoverable and reusable macroscopic alumina supported Ni-based catalyst for efficient hydrogen production. Sci. Rep..

[51] Nikoo MK, Amin NAS (2011). Thermodynamic analysis of carbon dioxide reforming of methane in view of solid carbon formation. Fuel Process. Technol..

[52] Barroso-Quiroga MM, Castro-Luna AE (2010). Catalytic activity and effect of modifiers on Ni-based catalysts for the dry reforming of methane. Int. J. Hydrogen Energy.

[53] Guo J, Lou H, Zhao H, Chai D, Zheng X (2004). Dry reforming of methane over nickel catalysts supported on magnesium aluminate spinels. Appl. Catal. A Gen..

[54] Chein RY, Fung WY (2019). Syngas production via dry reforming of methane over CeO_2_ modified Ni/Al_2_O_3_ catalysts. Int. J. Hydrogen Energy.

[55] Hu H, Ding W, Sun G, Yao Z (2022). Novel highly active and stable alumina-supported cobalt nitride catalyst for dry reforming of methane. Appl. Surf. Sci..

[56] Khavarian M, Chai SP, Mohamed AR (2015). The effects of process parameters on carbon dioxide reforming of methane over Co–Mo–MgO/MWCNTs nanocomposite catalysts. Fuel.

[57] Zhang Y (2023). Dry reforming of methane over Ni/SiO_2_ catalysts: Role of support structure properties. Fuel.

[58] Li B, Luo Y, Li B, Yuan X, Wang X (2019). Catalytic performance of iron-promoted nickel-based ordered mesoporous alumina FeNiAl catalysts in dry reforming of methane. Fuel Process. Technol..

[59] Rahbar Shamskar F, Rezaei M, Meshkani F (2017). The influence of Ni loading on the activity and coke formation of ultrasound-assisted co-precipitated Ni–Al_2_O_3_ nanocatalyst in dry reforming of methane. Int. J. Hydrogen Energy.

[60] Aghaali MH, Firoozi S (2019). Synthesis of nanostructured fcc/hcp hollow Ni particles by ultrasonic spray pyrolysis and its dry reforming catalytic properties. Powder Technol..

[61] Qiu H, Ran J, Huang X, Ou Z, Niu J (2022). Unrevealing the influence that preparation and reaction parameters have on Ni/Al_2_O_3_ catalysts for dry reforming of methane. Int. J. Hydrogen Energy.

