# Supplementary material for: Role of support bio-templating in Ni/Al2O3 catalysts for hydrogen production via dry reforming of methane
Source: Sci Rep. 2023 Oct 9;13:16972. doi: 10.1038/s41598-023-43782-8 (PMC10562384; doi:10.1038/s41598-023-43782-8)
Supplement: Supplementary file 1 — Supplementary Information 1. [file 41598_2023_43782_MOESM1_ESM.docx]

**Supplementary Information**

**Role of Support Bio-templating in Ni/Al_2_O_3_ Catalysts for Hydrogen Production**

**Via Dry Reforming of Methane**

Tayebeh Roostaei, Mohammad Reza Rahimpour^[[1]](#footnote-1)^

Department of Chemical Engineering, Shiraz University, Shiraz, Iran


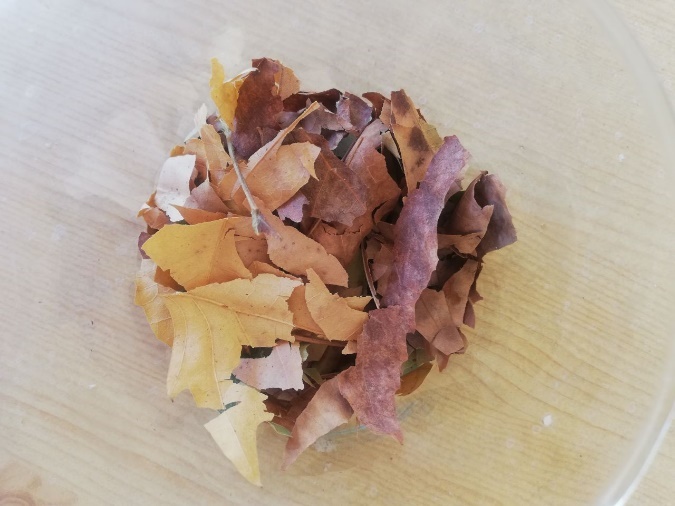


Figure S1- Optical image of used leaves.


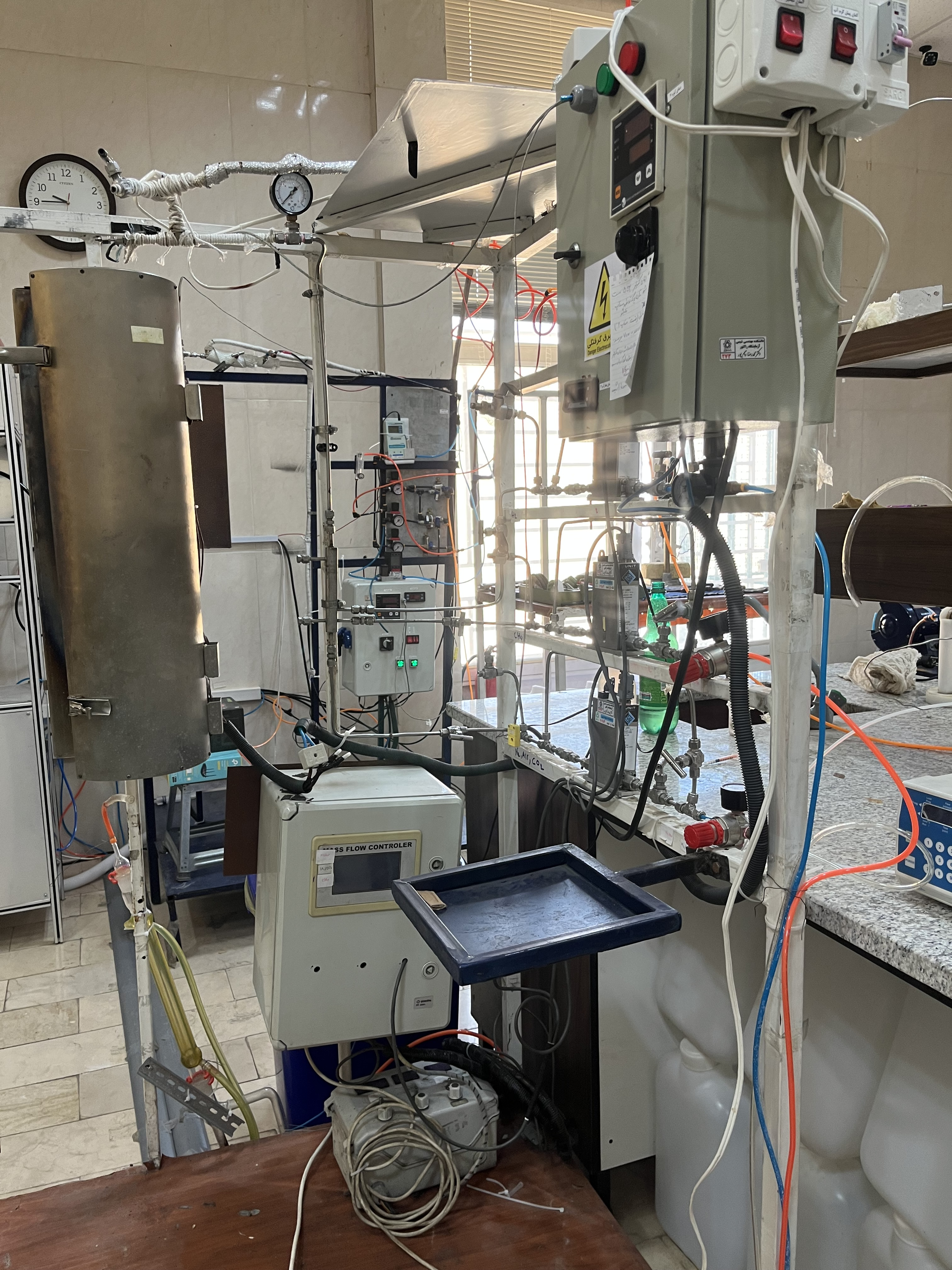

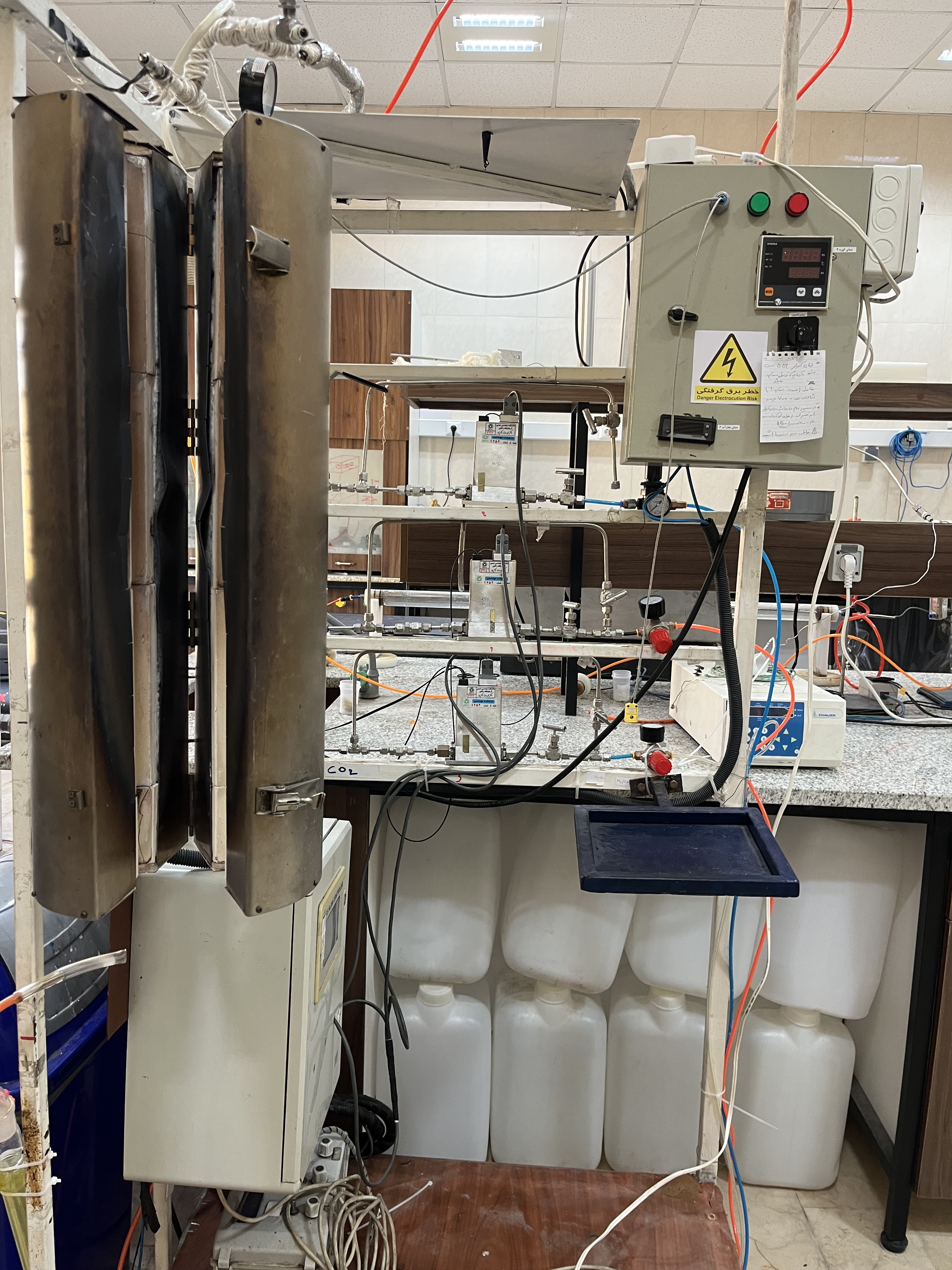


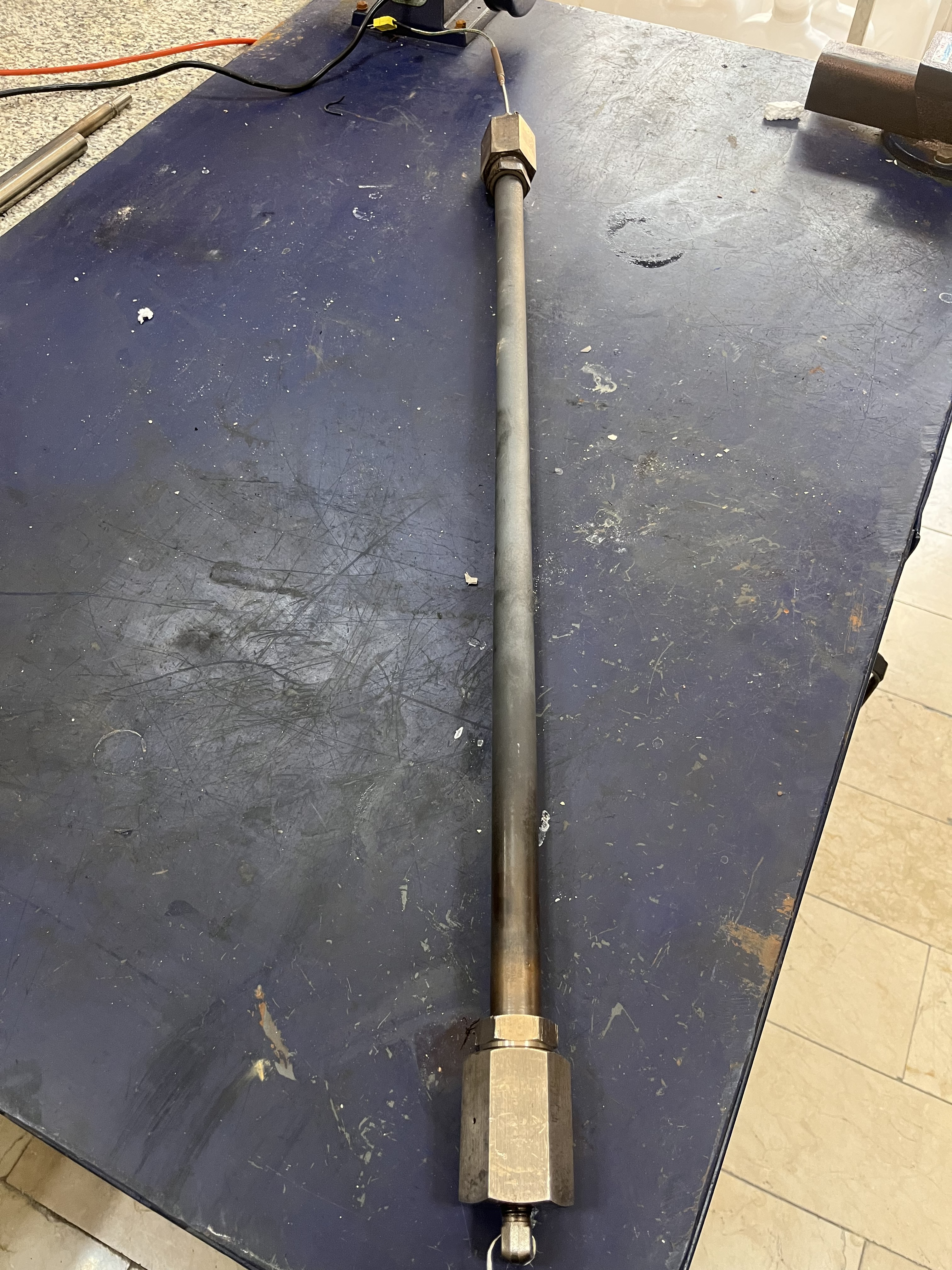

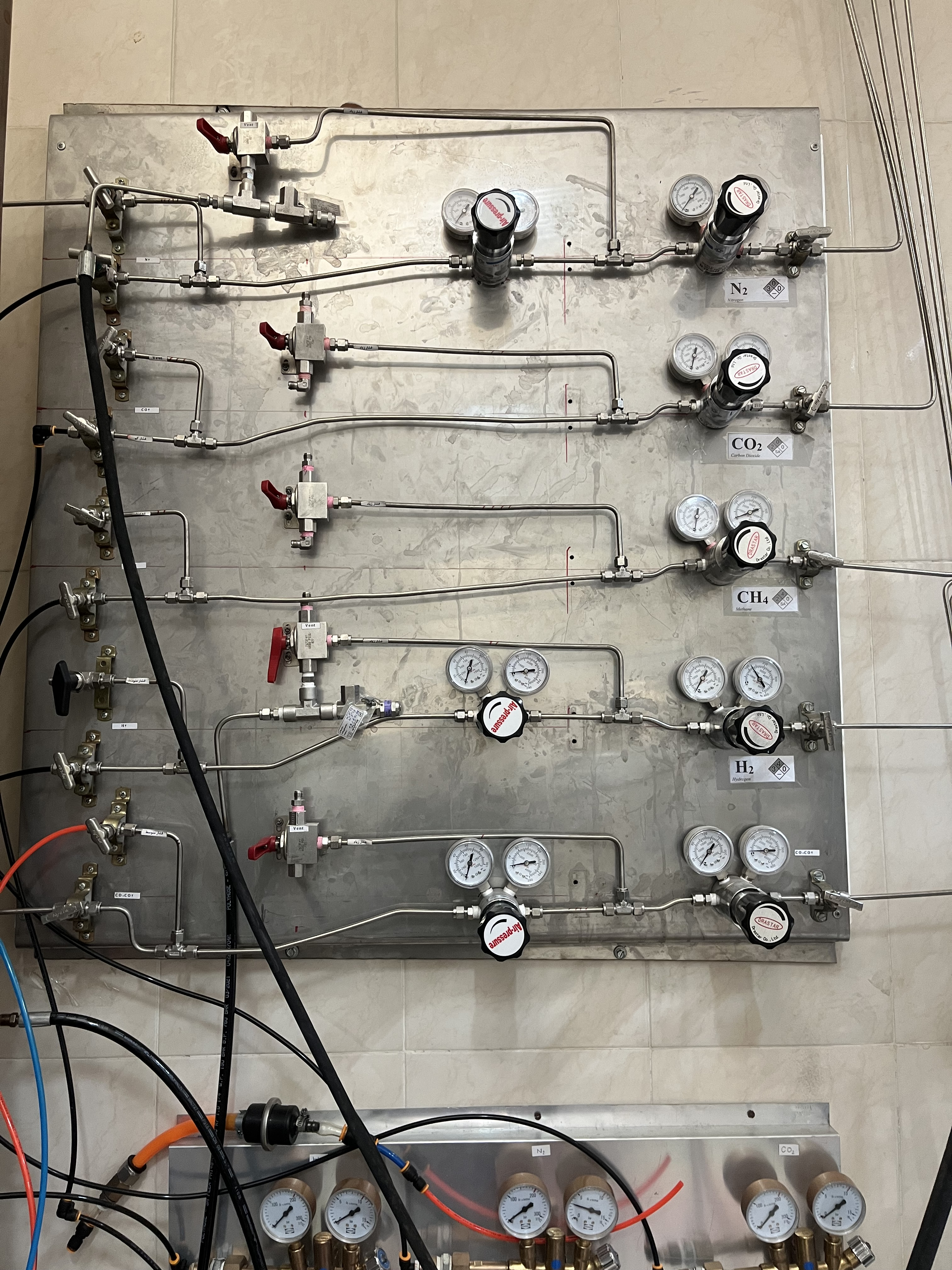


Figure S2- The set-up used for DRM experiments.


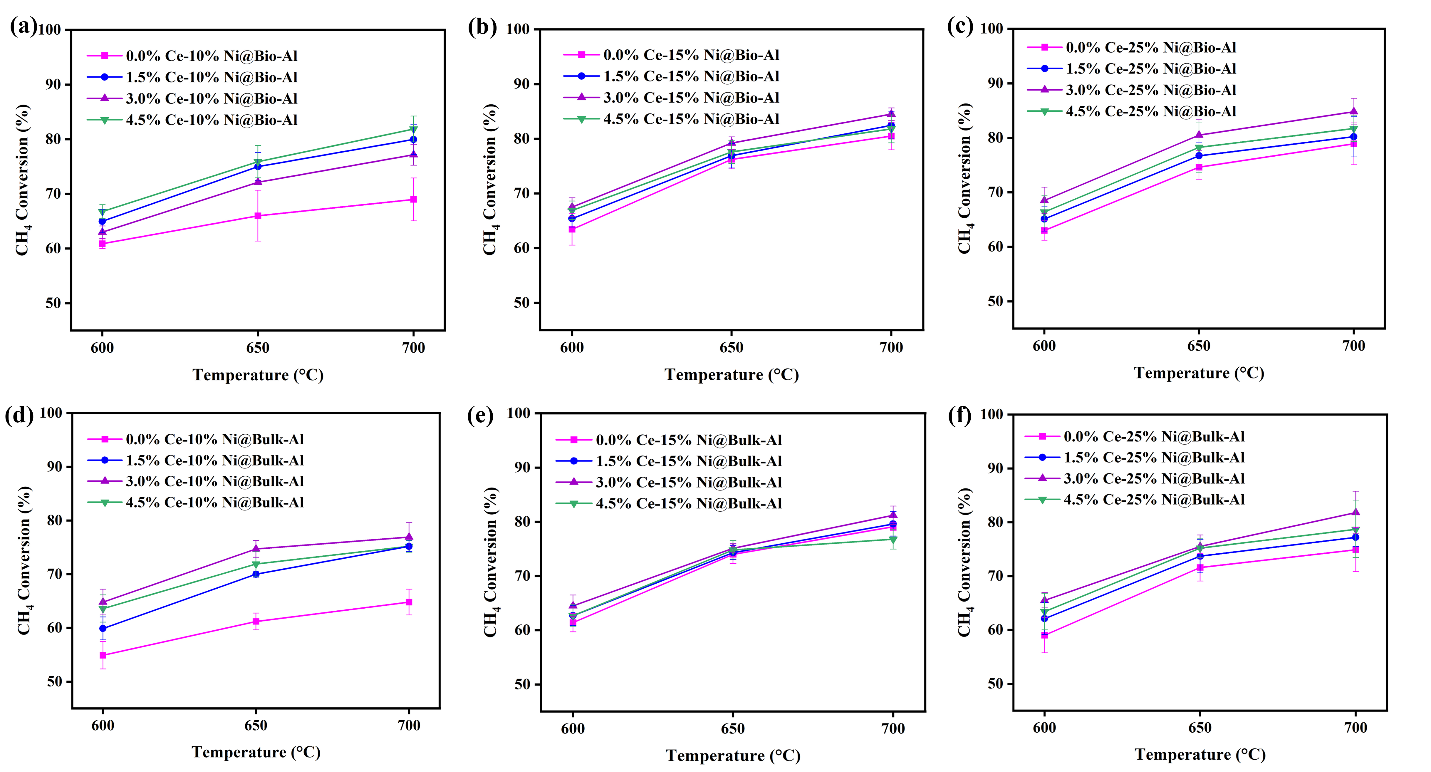


Figure S3. CH_4_ conversion for bio-templated and non-templated catalysts with different Ni and Ce loading amounts.


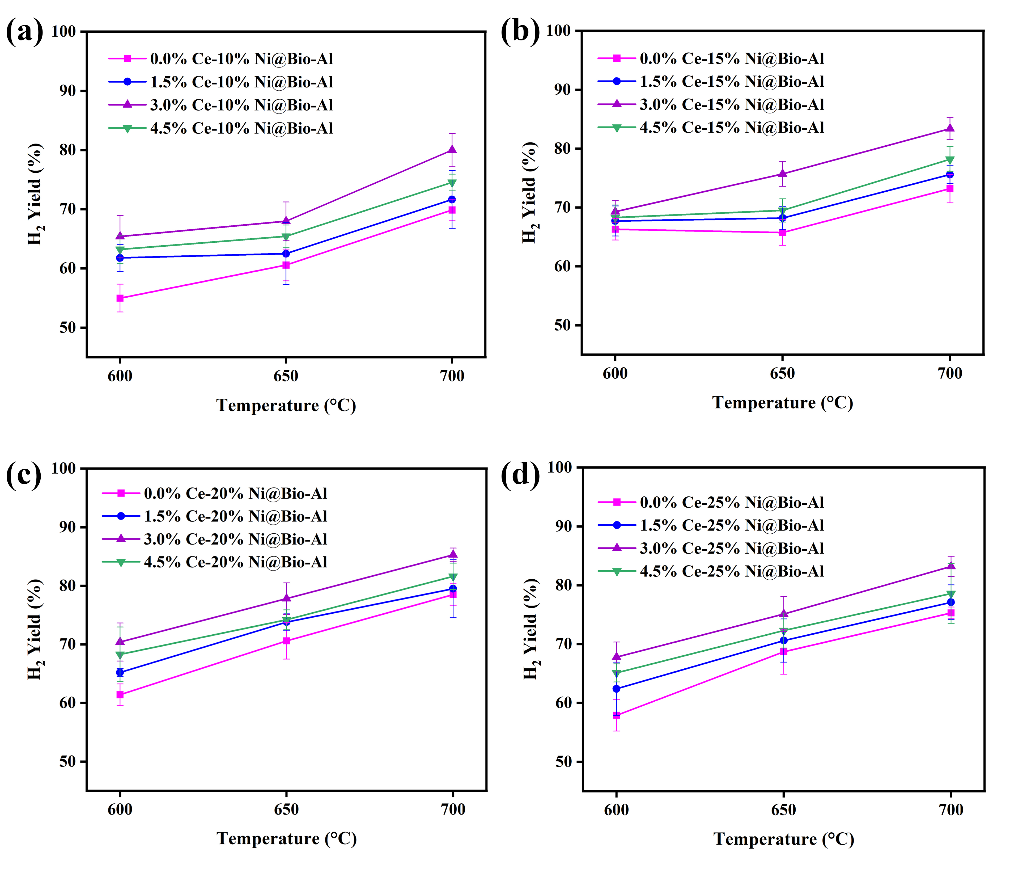


Figure S4. H_2_ yield for bio-templated catalysts with different Ni and Ce loading amounts.


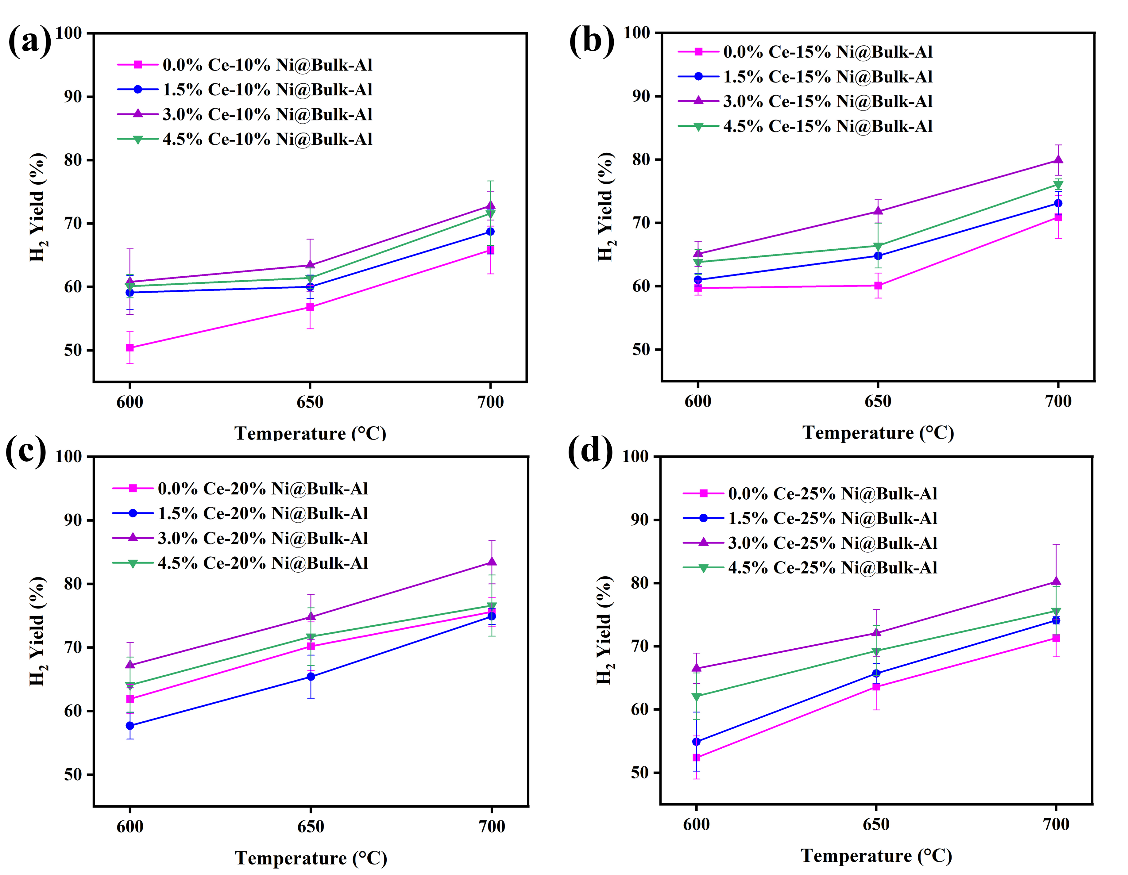


Figure S5. H_2_ yield for non-templated catalysts with different Ni and Ce loading amounts.

Table S1- GC results related to the gaseous products measurement.

| Sample | T (°C) | CO_2_ | CH_4_ | H_2_ | CO |
| --- | --- | --- | --- | --- | --- |
| 10%Ni@Bio-Al | 600 | 0.226921 | 0.132511 | 0.372182 | 0.268386 |
|  | 650 | 0.195816 | 0.110593 | 0.39392 | 0.299671 |
|  | 700 | 0.176239 | 0.095396 | 0.429743 | 0.298622 |
| 10%Ni@Bulk-Al | 600 | 0.2427 | 0.157602 | 0.344908 | 0.254791 |
|  | 650 | 0.208062 | 0.130154 | 0.368658 | 0.293126 |
|  | 700 | 0.202751 | 0.112337 | 0.411371 | 0.273542 |
| 15%Ni@Bio-Al | 600 | 0.200188 | 0.115171 | 0.417831 | 0.266809 |
|  | 650 | 0.165157 | 0.074902 | 0.414486 | 0.345455 |
|  | 700 | 0.149527 | 0.058959 | 0.442423 | 0.349091 |
| 15%Ni@Bulk-Al | 600 | 0.226523 | 0.127369 | 0.393987 | 0.25212 |
|  | 650 | 0.199512 | 0.085784 | 0.396586 | 0.318118 |
|  | 700 | 0.169711 | 0.064491 | 0.43755 | 0.328248 |
| 20%Ni@Bio-Al | 600 | 0.178237 | 0.110914 | 0.393648 | 0.317202 |
|  | 650 | 0.144473 | 0.069083 | 0.429716 | 0.356728 |
|  | 700 | 0.127235 | 0.056118 | 0.456501 | 0.360147 |
| 20%Ni@Bulk-Al | 600 | 0.189043 | 0.120126 | 0.396576 | 0.294255 |
|  | 650 | 0.168074 | 0.077576 | 0.432207 | 0.322144 |
|  | 700 | 0.14209 | 0.072709 | 0.446891 | 0.33831 |
| 25%Ni@Bio-Al | 600 | 0.190886 | 0.133755 | 0.377777 | 0.297582 |
|  | 650 | 0.153621 | 0.087115 | 0.421463 | 0.337802 |
|  | 700 | 0.138709 | 0.074123 | 0.44474 | 0.342427 |
| 25%Ni@Bulk-Al | 600 | 0.212837 | 0.125529 | 0.358458 | 0.303176 |
|  | 650 | 0.167929 | 0.080951 | 0.405393 | 0.345726 |
|  | 700 | 0.15149 | 0.06434 | 0.434831 | 0.349339 |

Table S2- Elemental composition of 3%Ce-20%Ni@Bio-Al and 3%Ce-20%Ni@Bulk-Al catalysts using ICP analysis.

| Sample | Ni (wt%) | Ce (wt%) |
| --- | --- | --- |
| 3%Ce-20%Ni@Bio-Al | 12.76 | 1.64 |
| 3%Ce-20%Ni@Bulk-Al | 11.42 | 1.78 |

1. Corresponding author: Rahimpor@shirazu.ac.ir [↑](#footnote-ref-1)
